# New Furan and Cyclopentenone Derivatives from the Sponge-Associated Fungus *Hypocrea Koningii* PF04

**DOI:** 10.3390/md13095579

**Published:** 2015-08-26

**Authors:** Li-Jian Ding, Bin-Bin Gu, Wei-Hua Jiao, Wei Yuan, Ying-Xin Li, Wei-Zhuo Tang, Hao-Bing Yu, Xiao-Jian Liao, Bing-Nan Han, Zhi-Yong Li, Shi-Hai Xu, Hou-Wen Lin

**Affiliations:** 1 College of Pharmacy, Jinan University, Guangzhou 510632, China; E-Mails: huahua20062008@126.com (L.-J.D.); tliaoxj@jnu.edu.cn (X.-J.L.); 2 Marine Drugs Research Center, State Key Laboratory of Oncogenes and Related Genes, Department of Pharmacy, Ren Ji Hospital, School of Medicine, Shanghai Jiao Tong University, Shanghai 200127, China; E-Mails: zuodiyake@outlook.com (B.-B.G.); weihuajiao@hotmail.com (W.-H.J.); yuanweiwork2013@163.com (W.Y.); tweizhuo@126.com (W.-Z.T.); yuhaobing1986@126.com (H.-B.Y.); bingnanh@hotmail.com (B.-N.H.); 3 State Key Laboratory of Microbial Metabolism, Marine Biotechnology Laboratory, School of Life Sciences and Biotechnology, Shanghai Jiao Tong University, Shanghai 200240, China; E-Mail: liyingxinhappy@126.com

**Keywords:** *Hypocrea koningii*, sponge-associated fungus, furan derivatives, cyclopentenone derivatives, antibacterial, antioxidant

## Abstract

Two new furan derivatives, hypofurans A and B (**1** and **2**), and three new cyclopentenone derivatives, hypocrenones A–C (**3**–**5**), along with seven known compounds (**6**–**12**), were isolated from a marine fungus *Hypocrea koningii* PF04 associated with the sponge *Phakellia fusca*. Among them, compounds **10** and **11** were obtained for the first time as natural products. The planar structures of compounds **1**–**5** were elucidated by analysis of their spectroscopic data. Meanwhile, the absolute configuration of **1** was determined as 2*R*,3*R* by the comparison of the experimental and calculated electronic circular dichroism (ECD) spectra. All the isolates were evaluated for their antibacterial and antioxidant activity. Compounds **1**, **10**, and **12** all showed modest antibacterial activity against *Staphylococcus aureus* ATCC25923 (MIC, 32 μg/mL). In addition, compounds **1**, **10** and **11** exhibited moderate DPPH radical scavenging capacity with IC_50_ values of 27.4, 16.8, and 61.7 µg/mL, respectively.

## 1. Introduction

Marine fungi harbor the potential to generate a multitude of structurally novel chemicals with diverse biological activities in part owing to harsh habitats [1,2]. More than one thousand new natural products have hitherto been harvested from marine-derived fungi. Excitingly, some of them with clinically relevant pharmacological activities will be probably developed into viable drug candidates exemplified by halimide, which entered into phase II clinical trials of cancer chemotherapy [3,4,5,6]. As an epitome of particular marine habitats, sponges often possess remarkable microbial biomass and diversity, and their microbial symbionts represent a precious wellspring of new scaffolds for drug discovery [7]. Hence, we focused on the sponge-derived fungus *Hypocrea koningii* PF04, which was associated with the marine sponge *Phakellia fusca* collected from Yongxing Island in the South China Sea [8].

**Figure 1:**
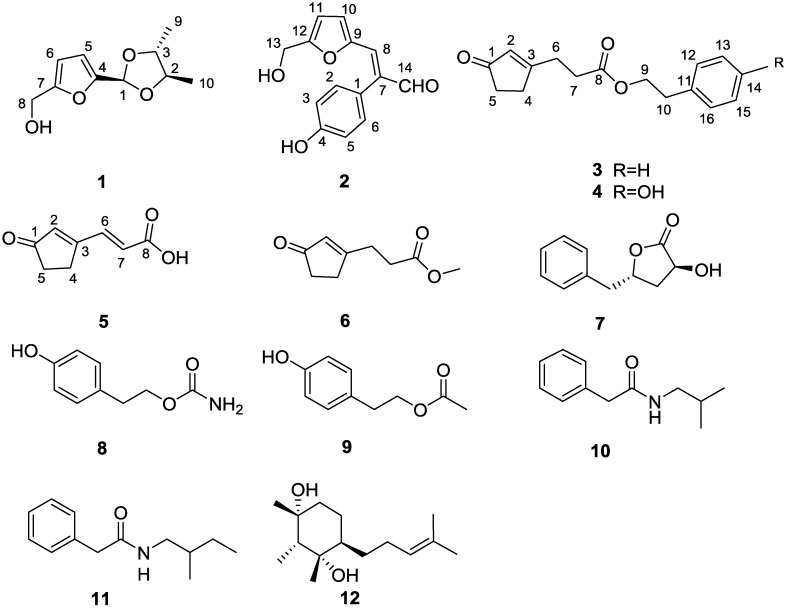
Chemical structures of compounds **1**–**12**.

Members of the genus *Hypocrea* (also called *Trichoderma*), typically soil-borne or wood-decaying fungi, are renowned for opportunistic pathogens of immunocompromised humans as well as producers of industrial enzymes and biocontrol agents against plant pathogens [9,10]. According to previous reports on the chemical constituents of this genus, various types of novel secondary metabolites, including terpenoids [11], polyketides [12,13], alkaloids [14], peptides [15], have been documented. Notably, a large proportion of these metabolites exhibited therapeutic properties such as antimalarial [12,16], tyrosine kinase inhibitory [13], antimicrobial [11,14,15], and cytotoxic activities [12]. In our present search for bioactive constituents from *H. koningii* PF04, two new furan derivatives, hypofurans A and B (**1** and **2**) and three new cyclopentenone derivatives, hypocrenones A–C (**3**–**5**), together with seven known compounds, methyl-3-(3-oxocyclopent-1-enyl) propionate (**6**), harzialactone A (**7**), tyrosol carbamate (**8**), tyrosyl acetate (**9**), *N*-isobutyl-2-phenylacetamide (**10**), *N*-(2-methylbutyl)-2-phenylacetamide (**11**), and citrantifidiol (**12**) (Figure 1) were isolated from the ethyl acetate (EtOAc) extract of the solid-state culture. Among them, new compounds **1** and **2** are furan derivatives, and **3**–**6** encompass cyclopentenone subunits. Both furan and cyclopentenone moieties are prevalent in natural products and pharmaceuticals [17,18]. Herein, we described the details of the isolation, structure elucidation, possible biosynthetic pathways, and biological activities of these compounds.

## 2. Results and Discussion

### 2.1. Structure Elucidation

Hypofuran A (**1**) was afforded as a colorless oil. The molecular formula C_10_H_14_O_4_ was deduced from HRESIMS data (*m*/*z* 199.0965 for [M + H]^+^), indicative of four degrees of unsaturation. The IR spectrum of **1** showed characteristic absorptions for a hydroxy group (3386 cm^−1^) and a furan ring (3123, 1584, 1522, 888 cm^−1^) [19]. The ^1^H NMR spectrum (Table 1) showed a 2,5-disubstituted furan ring at δ_H_ 6.42 (d, H-5) and 6.24 (d, H-6), one acetal methine at δ_H_ 5.89 (s, H-1), an exchangeable proton at δ_H_ 5.23 (t, OH-8), one hydroxymethyl group at δ_H_ 4.37 (d, H-8), two oxygenated methines at δ_H_ 3.70 (m, H-3) and 3.67 (m, H-2), and two methyls at δ_H_ 1.27 (d, H-10) and 1.22 (d, H-9). The ^13^C NMR and DEPT data (Table 2) displayed 10 carbons, including two olefinic quaternary carbons, five methines (two olefinic ones), one oxymethylene, and two methyls. The hydroxymethyl group was located at C-7 (δ_C_, 155.9) in the furan ring on the basis of HMBC correlations from H_2_-8 to C-6 and C-7. The hydroxymethylfuran ring accounted for three of four degrees of unsaturation, thus requiring an additional ring for **1**. The remaining ring was assigned as a dioxolane ring from the HMBC correlations of H-1/C-2 and C-3 and COSY correlation of H-2/H-3. A closer examination of the COSY spectrum implied that two methyls were tethered to C-2 and C-3 of the dioxolane ring, respectively, which was confirmed by correlations of H-2/H_3_-10 and H-3/H_3_-9. Further HMBC correlations of H-1/C-4 and C-5 established the connection of the dioxolane ring to C-4 of the hydroxymethylfuran ring. Accordingly, the gross structure of hypofuran A (**1**) was elucidated as shown in Figure 1.

**Table 1:** ^1^H NMR Data (600 MHz, DMSO-*d*_6_) for Compounds **1**–**5** (*J* in Hz).

Position	1	2	3	4	5
**1**	5.89, s				
**2**	3.67, m	6.99, d (8.5)	5.91, t (1.6)	5.87, s	6.46, s
**3**	3.70, m	6.82, d (8.5)			
**4**			2.56, m	2.53, m	2.76, m
**5**	6.42, d (3.2)	6.82, d (8.5)	2.39, m	2.27, m	2.41, m
**6**	6.24, d (3.1)	6.99, d (8.5)	2.69, t (7.3)	2.61, m	7.61, d (15.9)
**7**			2.60, t (7.3)	2.61, m	6.40, d (15.9)
**8**	4.37, d (5.4)	7.40, s			
**9**	1.22, d (5.8)		4.33, t (7.0)	4.17, t (6.9)	
**10**	1.27, d (5.8)	6.19, d (3.5)	2.95, t (7.0)	2.76, t (6.9)	
**11**		6.37, d (3.5)			
**12**			7.21, d (7.0)	7.02, d (8.4)	
**13**		4.36, d (4.9)	7.31, t (7.5)	6.68, d (8.4)	
**14**		9.63, s	7.24, t (7.5)		
**15**			7.31, t (7.5)	6.68, d (8.4)	
**16**			7.21, d (7.0)	7.02, d (8.4)	
**4**-OH		9.61, s			
**8**-OH	5.23, t (5.8)				12.8, s
**13**-OH		5.34, t (5.7)			
**14**-OH				9.24, s	

**Table 2:** ^13^C NMR (150 MHz, DMSO-*d*_6_) Data for Compounds **1**–**5**.

Carbon	1	2	3	4	5
**1**	95.8, CH	123.5, qC	209.6, qC	208.7, qC	208.7, qC
**2**	79.2, CH	130.3, CH	129.4, CH	128.4, CH	135.1, CH
**3**	77.5, CH	115.3, CH	180.3, qC	181.6, qC	169.3, qC
**4**	150.7, qC	157.4, qC	31.6, CH_2_	31.0, CH_2_	26.6, CH_2_
**5**	109.4, CH	115.3, CH	35.2, CH_2_	34.9, CH_2_	34.7, CH_2_
**6**	107.3, CH	130.3, CH	28.4, CH_2_	28.0, CH_2_	137.9, CH
**7**	155.9, qC	137.7, qC	31.5, CH_2_	30.9, CH_2_	126.4, CH
**8**	55.6, CH_2_	136.0, CH	172.0, qC	171.9, qC	166.7, qC
**9**	16.4, CH_3_	149.6, qC	65.2, CH_2_	64.9, CH_2_	
**10**	16.5, CH_3_	117.0, CH	35.0, CH_2_	33.5, CH_2_	
**11**		110.1, CH	137.5, qC	127.8, qC	
**12**		159.0, qC	128.8, CH	129.7, CH	
**13**		55.8, CH_2_	128.5, CH	115.1, CH	
**14**		193.4, CH	126.7, CH	155.9, qC	
**15**			128.5, CH	115.1, CH	
**16**			128.8, CH	129.7, CH	

**Figure 2:**
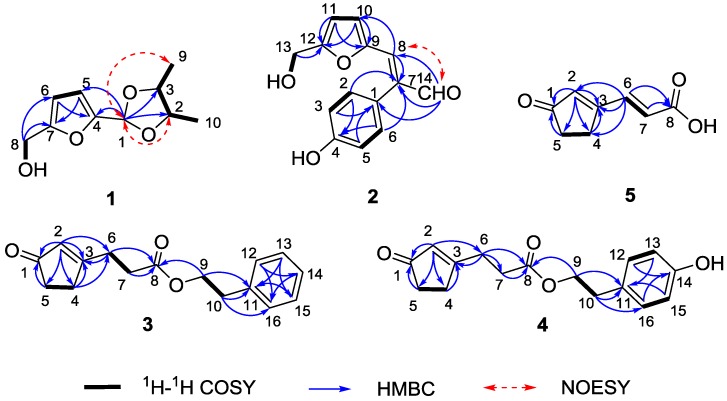
Key COSY, HMBC, and NOESY correlations of compounds **1**–**5**.

**Figure 3:**
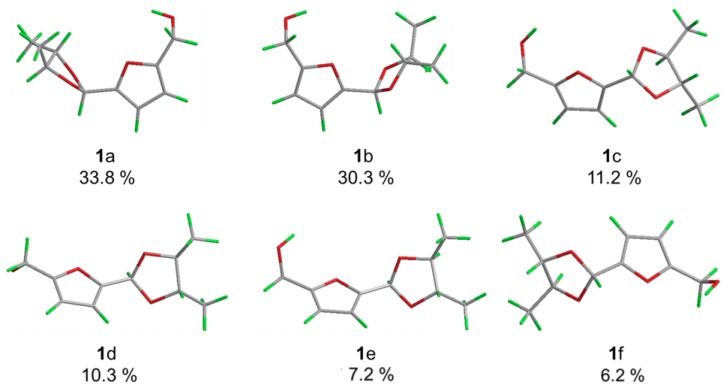
Low energy conformations of **1**.

**Figure 4:**
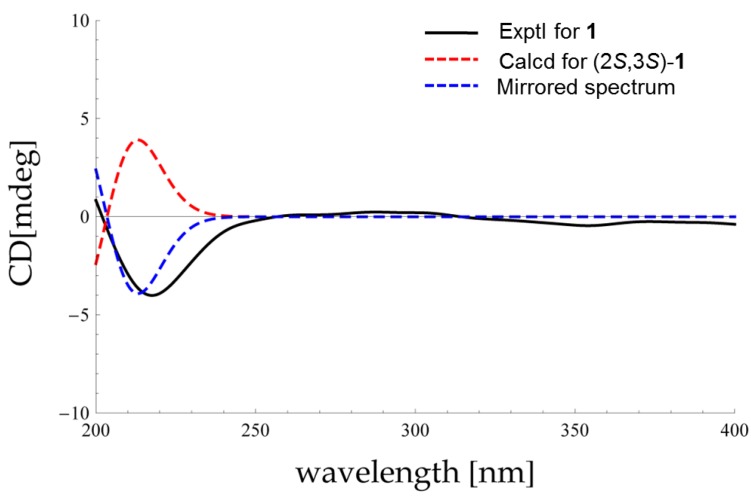
Comparison of experimental electronic circular dichroism (ECD) and calculated (MeOH) ECD spectra of **1**.

The relative stereochemistry of **1** was disclosed by a NOESY experiment (Figure 2). Two intense NOE interactions between H-1/H_3_-9 and H-1/H-2 suggested that H-1, H-2, and H_3_-9 were in the same orientation of the dioxolane ring while H-3 and H_3_-10 were cofacial. Thus, the relative configurations at C-2 and C-3 of **1** were assigned as 2*S*,3*S* or 2*R*,3*R*, respectively. In order to assign the absolute configuration of **1**, we chose to compare its experimental electronic circular dichroism (ECD) spectrum with the correspondingly time-dependent density functional theory (TDDFT) calculated one, which has proved to be a powerful and reliable approach for determining the absolute configuration of natural products [20,21]. Six low energy conformers above 1% population were generated using conventional initial Merck Molecular Force Field (MMFF) and DFT geometry optimization method (Figure 3 and Supplementary Information), for which ECD spectra were calculated with the TZVP basis set and four different functionals (B3LYP, BH&HLYP, PBE0, CAM-B3LYP). The experimental ECD spectrum (MeOH) of **1** showed a strong negative Cotton effect around 218 nm. With all four methods, the mirror image curve of the Boltzmann-averaged ECD spectrum of (2*S*,3*S*)-**1** was in good accordance with the experimental one, thus suggesting a (2*R*,3*R*) absolute configuration (Figure 4).

Hypofuran B (**2**) was furnished as a yellow powder. The HRESIMS data (*m*/*z* 267.0634 for [M + Na]^+^) supported a molecular formula of C_14_H_12_O_4_. The IR absorption bands at 3404 cm^−1^ (a hydroxy group), together with 3020, 1614, 1516, 905 cm^−1^ (a furan ring), implying **2** possessed a hydroxymethylfuran ring [19]. The assumption was further supported by comparison of its corresponding NMR spectra of **1**. The HMBC correlations of H-8 (δ_H_, 7.40)/C-7 (δ_C_, 137.7) and H-14 (δ_H_, 9.63)/C-7 and C-8 (δ_C_, 136.0) revealed that a formyl group (C-14, δ_C_, 193.4) was linked to a vinyl group (C-7–C-8), constructing an acrylaldehyde moiety. Furthermore, the remaining characteristic signals at δ_H_ 9.61 (s, 4-OH), 6.99 (d, 8.5 Hz, 2H), and 6.82 (d, 8.5 Hz, 2H) were assignable to a *para*-hydroxyphenyl ring, established by the HMBC correlations of H-2 and H-6/C-4. The connectivity of the hydroxymethylfuran ring, acrylaldehyde moiety and *para*-hydroxyphenyl ring were accomplished by analysis of the HMBC spectrum. The crucial cross-peak of H-8/C-10 (δ_C_, 117.0) clearly suggested that the hydroxymethylfuran ring was connected to the acrylaldehyde moiety, which was attached to the *para*-hydroxyphenyl ring based on the correlations of H-14/C-1 (δ_C_, 123.5) as well as H-2 and H-6/C-7 in the HMBC spectrum. The geometry of the double bond (C-7–C-8) was determined as *E* by the NOESY correlation between H-14 and H-8 (Figure 2). According to the aforementioned information, the structure of **2** was unambiguously assigned as depicted in Figure 1.

Hypocrenone A (**3**) appeared as a colorless oil. It was assigned a molecular formula of C_16_H_18_O_3_, based on HRESIMS data for *m*/*z* 259.1329 [M + H]^+^. The IR spectrum indicated the presence of two carbonyl groups (1735, 1707 cm^−1^) and a benzene ring (1676 cm^−1^). Interpretation of the ^13^C NMR spectrum disclosed the existence of a conjugated ketone carbonyl carbon (δ_C_ 209.6, C-1), an ester carbonyl carbon (δ_C_ 172.0, C-8), two conjugated olefinic carbons (δ_C_ 180.3, C-3 and 129.4, C-2), a monosubstituted phenyl ring [δ_C_ 137.5 (C-11), 128.8 (C-12, C-16), 128.5 (C-13, C-15), and 126.7 (C-14)], an oxygenated methylene carbon (δ_C_ 65.2, C-9), and four methylene groups [δ_C_ 35.2 (C-5), 35.0 (C-10), 31.6 (C-4), and 28.4 (C-6)] (Table 2). The COSY cross-peak of H-4/H-5 in conjunction with HMBC correlations of H-2/C-1, C-3, C-4, and C-5, H-4/C-3 and C-8, and H_2_-5/C-1, C-3, and C-4 delineated a cyclopentenone moiety. Furthermore, the COSY cross-peaks of H_2_-6/H_2_-7 and H_2_-9/H_2_-10 suggested the presence of two spin systems, which were connected through an ester bond based on the HMBC correlations from H_2_-6, H_2_-7, and H_2_-9 to C-8 (Figure 2). Finally, C-6 was adjacent to the cyclopentenone moiety, as evident by the HMBC cross peaks of H-2 and H-4/C-6 and H-6/C-3, while C-10 was linked to the benzene ring, as indicated by the HMBC correlations of H-9 and H-10/C-11 and H-10/C-16, thereby establishing the structure of **3**.

Hypocrenone B (**4**) was obtained as a colorless oil. Its molecular formula C_16_H_18_O_4_, was evidenced by HRESIMS data (*m*/*z* 297.1104 for [M + Na]^+^), which was one oxygen atom more than **3**. The ^1^H NMR spectrum of **4** resembled that of compound **3** except for the presence of an exchangeable proton (δ_H_, 9.24) and the conspicuous absence of an aromatic proton of **3** (Table 1). Further scrutiny of 1D NMR data, two sets of *ortho*-coupled aromatic proton signals [δ_H_ 7.02 and 6.68 (2H each, d, *J* = 8.4 Hz)] and six aromatic carbon signals, including an oxygenated one [δ_C_ 155.9 (C-14), 129.7 (C-12, C-16), 127.8 (C-11), and 115.1 (C-13, C-15)] were observed, indicating the presence of a *para*-hydroxyphenyl moiety in **4** instead of the phenyl ring in **3**. The structure of **4** was further established by 2D NMR data (Figure 2) and named hypocrenone B.

Hypocrenone C (**5**) was yielded as a white powder. The HRESIMS spectrum of **5** exhibited a pseudomolecular ion peak at *m*/*z* 151.0394 [M − H]^−^, corresponding to its molecular formula C_8_H_8_O_3_. Diagnostic NMR data for **5** suggested the presence of a cyclopentenone moiety, identical with that of **3** and **4**. Moreover, the COSY correlation of H-6 (δ_H_ 7.61)/H-7 (δ_H_ 6.40) and HMBC correlations from H-6 and H-7 to C-8 (δ_C_ 166.7), together with IR absorption band at 1714 cm^−1^ (carboxylic acid) allowed the unambiguous assignment of the acrylic acid moiety, which was adjacent to the C-3 of the cyclopentenone moiety. The connectivity was corroborated by a significant upfield shift for C-3 (δ_C_ 169.3, Δδ = -12.3) and the HMBC correlations of H-6/C-2 (δ_C_ 135.1), C-3 (δ_C_ 169.3), and C-4 (δ_C_ 26.6) (Figure 2). The *E*-configuration of the C-6/C-7 double bond was inferred from the large coupling constant value (15.9 Hz).

In addition, seven known compounds were identified as methyl-3-(3-oxocyclopent-1-enyl) propionate (**6**) [22], harzialactone A (**7**) [23], tyrosol carbamate (**8**) [24], tyrosyl acetate (**9**) [25], *N*-isobutyl-2-phenylacetamide (**10**) [26], *N*-(2-methylbutyl)-2-phenylacetamide (**11**) [27] and citrantifidiol (**12**) [28] by analysis and comparison of their spectroscopic data with the literature. Among them, compounds **10** and **11** have been previously synthesized but this article is the first report of their isolation as natural products.

### 2.2. Plausible Biosynthetic Pathways

**Scheme 1:**
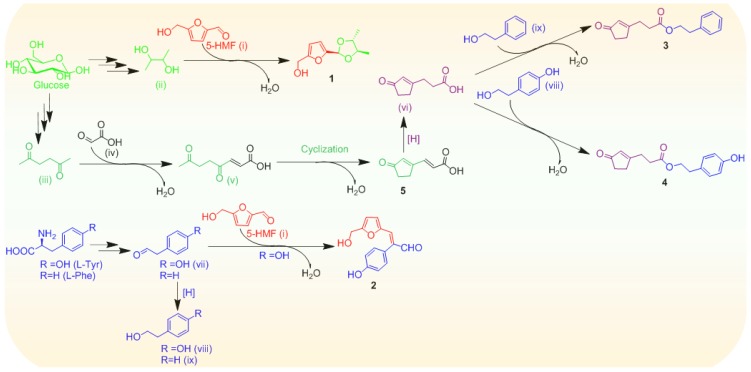
Plausible biosynthetic pathways of **1**–**5**.

Possible biogenetic routes to compounds **1**–**5** were proposed as shown in Scheme 1. The biosynthetic precursors, probably including 5-hydroxymethyl furfural (5-HMF, (i)), 2,3-butanediol (ii) and hexane-2,5-dione (iii) could originate from hexose sugars (e.g., glucose) [29,30,31]. The 5-HMF (i) could be ligated to 2,3-butanediol (ii), resultantly providing **1**. Hexane-2,5-dione (iii) might be condensed with glyoxylic acid (iv), followed by an aldol-type cyclization to form the cyclopentenone-containing metabolite **5**, the hydrogenation of which perhaps produced the intermediate (vi). Furthermore, a C6-C2 unit obtained from either l-tyrosine or l-phenylalanine, presumably acted as a building block for the new compound assembly lines. l-tyrosine underwent decarboxylation and deamination, thereby providing 4-hydroxyphenylacetaldehyde (vii), and the further hydrogenation of the C-6/C-7 double bond formed 4-hydroxyethylphenol (viii) [32]. Analogously, l-phenylalanine might be converted to 2-phenylethanol (ix) [32]. Subsequently, 4-hydroxyphenylacetaldehyde (vii) could proceed aldol condensation with 5-HMF (i) to afford **2**. The intermediates (ix) and (viii) were probably esterified with the substrate (vi) to yield **3** and **4**, respectively. Overall, both cyclopentenone and furan moieties were most likely derived from sugar precursors, together with a C6-C2 unit elaborated from either l-tyrosine or l-phenylalanine, presumably constructing the framework of the new compounds.

### 2.3. Biological Activity

All the isolates (**1**–**12**) were evaluated for antibacterial activities against Gram-positive *Staphylococcus aureus* ATCC25923 and methicillin-resistant *Staphylococcus aureus* (MRSA) ATCC43300 as well as Gram-negative *Escherichia coli* ATCC25922. The MIC values for the compounds **1**–**12** were no less than 64 μg/mL against the tested pathogens, except for **1**, **10** and **11**. These three compounds selectively inhibited the growth of *S. aureus* with the same MIC value of 32 μg/mL. Meanwhile, Compounds **1**–**12** were also tested for antioxidant activities using DPPH radical scavenging assay. The results (Table 3) showed that compounds **1**, **10** and **11** had moderate DPPH radical scavenging activities with IC_50_ values of 27.4 ± 7.4, 16.8 ± 4.3 and 61.7 ± 3.3 µg/mL, respectively, and the rest of the compounds did not show DPPH radical scavenging capacities (IC_50_ > 128 µg/mL).

**Table 3:** DPPH radical scavenging activity of compounds **1**–**12** (IC_50_, µg/mL).

Compound	IC_50_	Compound	IC_50_
**1**	27.4 ± 7.4	**8**	>128
**2**	>128	**9**	>128
**3**	>128	**10**	16.8 ± 4.3
**4**	>128	**11**	61.7 ± 3.3
**5**	>128	**12**	>128
**6**	>128	**Ascorbic acid**	4.4 ± 0.4
**7**	>128		

## 3. Experimental Section

### 3.1. General Experimental Procedures

Optical rotations were measured on a Perkin-Elmer model 341 polarimeter (Perkin-Elmer Inc., Waltham, MA, USA). UV data were performed on a Hitachi U-3010 spectrophotometer (Hitachi Inc., Tokyo, Japan). IR (KBr) spectra were carried out on a Jasco FTIR-400 spectrometer (Jasco Inc., Tokyo, Japan). ^1^H, ^13^C, and 2D NMR spectra were obtained on a Varian 600 MHz spectrometer (Palo Alto, CA, USA). CD spectra were collected using a Jasco J-715 spectropolarimeter (Jasco Inc., Tokyo, Japan). HRESIMS and ESIMS data were acquired on an Agilent Technologies 6224 TOF LC-MS apparatus (Agilent Technologies Co., Ltd, Beijing, China) and a Waters Q-Tof micro YA019 mass spectrometer (Waters Corp., Milford, MA, USA). Preparative medium pressure liquid chromatography (MPLC) was carried out on Puriflash 450 Instruments (Interchim Company, Montlucon, France). Semi-preparative reversed-phase HPLC (RP-HPLC) was performed on a YMC-Pack Pro C18 RS column (5 μm, 250 × 10 mm id; YMC, Kyoto, Japan) with a Waters 1525 separation module equipped with a Waters 2998 Photodiode Array (PDA) detector. Column chromatography (CC) was performed on silica gel (200–300 mesh, Qingdao Haiyang Chemical Factory, Qingdao, China). Flash chromatographic column (ODS, 15 μm, Santai Technologies, Inc., Changzhou, China) was used for MPLC. Thin-Layer chromatography (TLC) analysis was performed on silica gel HSGF 254 plates and visualized by spraying with 10% anisaldehyde-H_2_SO_4_ reagent.

### 3.2. Fungal Material

The fungus *H. koningii* PF04 was isolated from the tissue of the sponge *P. fusca* collected from Yongxing Island in the South China Sea. The fungus was identified by its rDNA amplification and sequence analysis of the ITS region (GenBank accession no. FJ941853). A voucher strain was deposited at the School of Life Sciences and Biotechnology, Shanghai Jiao Tong University, Shanghai, China.

### 3.3. Fermentation

The strain was initially grown on PDA medium in a Petri dish for 7 days. A single colony was inoculated into seed medium (potato 200 g, dextrose 20 g, sea water 1000 mL) in 250 mL Erlenmeyer flasks on a rotatory shaker (180 rpm) at 25 °C for 48 h. Subsequently, the large scale fermentation was carried out in 50 × 250 mL Erlenmeyer flasks (80 g of rice and 120 mL of sea water), each of which was inoculated with seed medium (10 mL). The fungus PF04 was cultured under static conditions at 25 °C for 40 days.

### 3.4. Extraction and Isolation

The fermented material was extracted with acetone (3 × 5 L). The organic solvent was concentrated under reduced pressure and partitioned with EtOAc (1.5 L) and H_2_O (1.5 L) to yield the EtOAc extract (24.5 g). The extract was subjected to vacuum liquid chromatography (VLC) on silica gel column (6 × 15 cm, 200–300 mesh) using petroleum ether/EtOAc (20:1, 10:1, 8:1, 5:1, 4:1, 3:1, 2:1, 1:1, 0:1, *v/v*, gradient) to generate seven fractions (A–E). Fraction C (1.1 g) was further separated by MPLC with a gradient of MeOH/H_2_O (from 10% to 100% MeOH, 180 min) to afford ten subfractions (C1–C10) and the resulting subfraction C3 was further purified by RP-HPLC eluting 35% MeCN/H_2_O at a flow rate of 2 mL/min, to afford **8** (2.0 mg, *t_R_* = 26.6 min), **7** (3.4 mg, *t_R_* = 36.6 min), **9** (7.2 mg, *t_R_* = 46.4 min), and **6** (2.5 mg, *t_R_* = 66.3 min). Compounds **12** (3.0 mg, *t_R_* = 16.6 min), **3** (4.0 mg, *t_R_* = 43.4 min), and **11** (1.2 mg, *t_R_* = 45.6 min) were obtained by RP-HPLC (2.0 mL/min, 40% MeCN/H_2_O) from fraction C5. Fraction C7 was purified by RP-HPLC with an elution of 65% MeCN (2.0 mL/min) to yield **10** (8.6 mg, *t_R_* = 22.6 min). Fraction D (3.75 g) was separated by MPLC on to give eleven fractions (D1–D11). Fraction D4 were isolated by RP-HPLC eluting with 10% MeCN to afford **5** (2.0 mL/min, 2.5 mg, *t_R_* = 28.5 min). Fraction D10 was further purified by RP-HPLC eluting with 35% MeCN to obtain **1** (5.0 mg, *t_R_* = 36.6 min) and **4** (3.0 mg, *t_R_* = 38.6 min). Fraction D5 was further purified by RP-HPLC (35% MeCN in H_2_O, 2.0 mL/min) to afford **2** (3.0 mg, *t_R_* = 26.9 min).

Hypofuran A (**1**): colorless oil; [α]${}_{D}^{25}$ +2.00 (*c* 0.50, MeOH); UV (MeOH) (log ε) λ_max_ 280 (4.68) nm; CD λ_max_ (Δε) 218 (−22.80) nm; IR (KBr) ν_max_ 3386, 3123, 2975, 2930, 2874, 2860, 1674, 1584, 1522, 1449, 1398, 1379, 1340, 1280, 1193, 1094, 1022, 989, 967, 888, 809, 777 cm^−1^; ^1^H and ^13^C NMR data, see Table 1 and Table 2; HRESIMS *m*/*z* 199.0965 [M + H]^+^ (calcd for C_10_H_15_O_4_, 199.0965).

Hypofuran B (**2**): yellow powder; UV (MeOH) (log ε) λ_max_ 337 (4.61); IR (KBr) ν_max_ 3404, 3020, 2959, 2934, 1717, 1614, 1516, 1447, 1435, 1396, 1358, 1310, 1262, 1224, 1173, 1087, 1044, 1009, 905, 831 cm^−1^; ^1^H and ^13^C NMR data, see Table 1 and Table 2; HRESIMS *m*/*z* 267.0634 [M + Na]^+^ (calcd for C_14_H_12_O_4_Na, 267.0633).

Hypocrenone A (**3**): colorless oil; UV (MeOH) (log ε) λ_max_ 235 (4.68), 288 (3.88), 341 (3.72); IR (KBr) ν_max_ 3441, 3064, 3029, 2959, 2926, 2860, 1735, 1707, 1676, 1616, 1496, 1435, 1339, 1282, 1240, 1174, 1090, 1053, 926, 846, 750, 701 cm^−1^; ^1^H and ^13^C NMR data, see Table 1 and Table 2; HRESIMS *m*/*z* 259.1329 [M + H]^+^ (calcd for C_16_H_19_O_3_, 259.1329).

Hypocrenone B (**4**): colorless oil; UV (MeOH) (log ε) λ_max_ 232 (4.69), 278 (4.39), 341 (4.06); IR (KBr) ν_max_ 3346, 3018, 2957, 2930, 2866, 1731, 1708, 1673, 1613, 1516, 1442, 1383, 1344, 1263, 1235, 1169, 1109, 1062, 1009, 833 cm^−1^; ^1^H and ^13^C NMR data, see Table 1 and Table 2; HRESIMS *m*/*z* 297.1104 [M + Na]^+^ (calcd for C_16_H_18_O_4_Na, 297.1103).

Hypocrenone C (**5**): white powder; UV (MeOH) (log ε) λ_max_ 272 (4.57); IR (KBr) ν_max_ 2925, 2858, 1714, 1654, 1625, 1569, 1513, 1434, 1398, 1356, 1294, 1266, 1232, 1102, 1045, 1018, 987, 928, 855, 825 cm^−1^; ^1^H and ^13^C NMR data, see Table 1 and Table 2; HRESIMS *m*/*z* 151.0394 [M − H]^−^ (calcd for C_8_H_7_O_3_, 151.0395).

### 3.5. Antibacterial Activity Assay

The antibacterial activities were evaluated against three different bacteria (*S. aureus* ATCC25923, methicillin-resistant *S. aureus* (MRSA) ATCC43300 and *E. coli* ATCC25922) in 96-well microplates as described by Correa with some modifications [33]. The final concentrations of each compound in the wells were 256, 128, 64, 32, 16, 8, 4 and 2 μg/mL. Chloramphenicol was used as positive control. Each assay was carried out in triplicates.

### 3.6. DPPH Radical Scavenging Activity Assay

The DPPH free radical scavenging assay was performed by a modified method [34]. Briefly, the tested compounds were dissolved in methanol to serial concentrations. Each well in 96-well microplates containing 100 μL of sample solution and 100 μL of DPPH solution (methanol, 0.2 mM) was incubated at 37 °C for 30 min. Ascorbic acid was used as positive control. The blank control experiment was added methanol without any dissolved compound. The absorbance was measured at 517 nm on a microplate reader. DPPH scavenging rate (%) = (1 − absorbance of compound/absorbance of control) × 100. All experiments were taken in triplicates. The IC_50_ value (the concentration of a compound to scavenge 50% of DPPH radicals) was calculated from nonlinear regression analysis using the GraphPad Prism software 5.0 (GraphPad Software, San Diego, USA). Results are presented as the means ± SD.

### 3.7. Theoretical ECD Calculations

All calculations were performed with the Gaussian 09 program using various functionals (B3LYP, BH&HLYP, PBE0, CAM-B3LYP) and TZVP basis set. See Supplementary Information for more details of the DFT calculation.

## 4. Conclusions

Chemical investigation of the sponge-associated fungus *Hypocrea koningii* PF04 has resulted in the isolation and characterization of five new compounds, hypofurans A and B (**1** and **2**) and hypocrenones A–C (**3**–**5**), along with seven known secondary metabolites (**6**–**12**) encompassing two new natural products (**10** and **11**), representing a paradigm of chemical diversity. Structurally, hypofurans A was a furan derivative featuring a dioxolane ring where the absolute configuration was ascertained by comparing its experimental and calculated ECD spectra. With respect to the biogenetic relationships of all the new compounds, the plausible avenues furnishing them were postulated. In a small panel of antibacterial assays, compounds **1**, **10** and **12** displayed modest inhibitory activities against *S. aureus* ATCC25923. In addition, compounds **1**, **10** and **11** exhibited moderate DPPH radical scavenging activity. Collectively, this article showcased marine fungi served as new bioactive secondary metabolite producers.

## Supplementary Files

Supplementary File 1
