# Supplementary material for: New Furan and Cyclopentenone Derivatives from the Sponge-Associated Fungus Hypocrea Koningii PF04
Source: Mar Drugs. 2015 Aug 26;13(9):5579–92. doi: 10.3390/md13095579 (PMC4584341; doi:10.3390/md13095579)
Supplement: Supplementary File 1 [file marinedrugs-13-05579-s001.doc]

Supplementary Information

S1. ECD Calculation Details

S1.1. Methods

Monte Carlo multiple minimum (MCMM) conformational searches were carried out by means of the Macromodel 9.9.223 [1] software using Merck Molecular Force Field (MMFF) applying a 21 kJ/mol energy window. Geometry reoptimizations of the resultant conformers (B3LYP/6-31G(d) level with PCM solvent model for CH_3_OH) and TDDFT calculations were performed with Gaussian 09 [2] using various functionals (B3LYP, BH&HLYP, PBE0, CAM-B3LYP) and TZVP basis set. The ECD spectra were generated using the program SpecDis [3] by applying a Gaussian band shape with the width of
0.3 eV, from dipole-length rotational strengths. Boltzmann distributions were estimated from the B3LYP/6-31G(d) free energies in the solvent model calculations. The Chem3D 8.0.3 software package was used for visualization of the results.

S1.2. Results

**Table S1.** Gibbs free energies ^a^ and equilibrium populations ^b^ of low-energy conformers of **1**.

| **Conformer** | **In MeOH** | |
| --- | --- | --- |
|  | **∆*G*** | **P (%)** |
| **Compound 1** |  |  |
| **1a** | 0.00 | 33.8 |
| **1b** | 6.53 × 10^−2^ | 30.3 |
| **1c** | 6.52 × 10^−1^ | 11.2 |
| **1d** | 7.03 × 10^−1^ | 10.3 |
| **1e** | 9.17 × 10^−1^ | 7.2 |
| **1f** | 1.00 | 6.2 |

^a^ B3LYP/6-31G(d), in kcal/mol; ^b^ From ∆*G* values at 298.15 K.

**Table S2.** Cartesian coordinates for the low-energy reoptimized MMFF conformers of **1** at B3LYP/6-31G(d) level of theory in CH_3_OH.

| **Compound 1  Conformer 1a** | | **Standard Orientation (Ångstroms)** | | |
| --- | --- | --- | --- | --- |
| I | Atom | X | Y | Z |
| 1 | 6 | 2.908590 | −1.301931 | −0.075124 |
| 2 | 6 | 2.550454 | −0.022094 | 0.234707 |
| 3 | 8 | 1.182314 | 0.091874 | 0.220855 |
| 4 | 6 | 0.675190 | −1.136783 | −0.107294 |
| 5 | 6 | 1.691689 | −2.025652 | −0.295186 |
| 6 | 6 | 3.314246 | 1.222275 | 0.541806 |
| 7 | 6 | −0.807140 | −1.265085 | −0.221713 |
| 8 | 8 | −1.425207 | −0.889838 | 1.001609 |
| 9 | 6 | −2.577144 | −0.089021 | 0.684375 |
| 10 | 6 | −2.138726 | 0.596938 | −0.612324 |
| 11 | 8 | −1.350531 | −0.427996 | −1.244105 |
| 12 | 6 | −3.253028 | 1.023487 | −1.550614 |
| 13 | 6 | −2.887687 | 0.826190 | 1.854320 |
| 14 | 8 | 3.274608 | 2.188247 | −0.515025 |
| 15 | 1 | 3.918754 | −1.682686 | −0.138132 |
| 16 | 1 | 1.586202 | −3.069249 | −0.558802 |
| 17 | 1 | 4.366540 | 0.961732 | 0.678417 |
| 18 | 1 | 2.951181 | 1.664337 | 1.480959 |
| 19 | 1 | −1.048418 | −2.305410 | −0.483098 |
| 20 | 1 | −3.433077 | −0.749917 | 0.473742 |
| 21 | 1 | −1.492999 | 1.454497 | −0.368521 |
| 22 | 1 | −2.839538 | 1.424132 | −2.481452 |
| 23 | 1 | −3.861037 | 1.807239 | −1.085665 |
| 24 | 1 | −3.900238 | 0.174368 | −1.794642 |
| 25 | 1 | −3.111798 | 0.240911 | 2.751567 |
| 26 | 1 | −3.760947 | 1.447583 | 1.628403 |
| 27 | 1 | −2.036111 | 1.480528 | 2.068746 |
| 28 | 1 | 2.353348 | 2.483688 | −0.595908 |

B3LYP Energy = −690.35077605 a.u.

**Table S2.** *Cont.*

| **Compound 1  Conformer 1b** | | **Standard Orientation (Ångstroms)** | | |
| --- | --- | --- | --- | --- |
| I | Atom | X | Y | Z |
| 1 | 6 | −2.891473 | −1.325043 | −0.178769 |
| 2 | 6 | −2.539083 | −0.094305 | 0.293698 |
| 3 | 8 | −1.176546 | 0.057089 | 0.219928 |
| 4 | 6 | −0.667012 | −1.098267 | −0.309313 |
| 5 | 6 | −1.676137 | −1.978565 | −0.564892 |
| 6 | 6 | −3.304650 | 1.074741 | 0.816304 |
| 7 | 6 | 0.813786 | −1.190542 | −0.469776 |
| 8 | 8 | 1.279383 | −0.138644 | −1.304370 |
| 9 | 6 | 2.481576 | 0.389502 | −0.717352 |
| 10 | 6 | 2.232061 | 0.161852 | 0.776077 |
| 11 | 8 | 1.506710 | −1.081094 | 0.774552 |
| 12 | 6 | 3.469008 | 0.019497 | 1.644283 |
| 13 | 6 | 2.659268 | 1.831687 | −1.154821 |
| 14 | 8 | −3.332403 | 2.184192 | −0.089011 |
| 15 | 1 | −3.896780 | −1.718205 | −0.243288 |
| 16 | 1 | −1.566941 | −2.971892 | −0.978526 |
| 17 | 1 | −2.900064 | 1.383789 | 1.791150 |
| 18 | 1 | −4.344672 | 0.774360 | 0.964606 |
| 19 | 1 | 1.058127 | −2.172839 | −0.898934 |
| 20 | 1 | 3.341493 | −0.219795 | −1.038378 |
| 21 | 1 | 1.582054 | 0.962239 | 1.161567 |
| 22 | 1 | 3.189868 | −0.223755 | 2.674255 |
| 23 | 1 | 4.032265 | 0.958965 | 1.659297 |
| 24 | 1 | 4.119737 | −0.774807 | 1.263383 |
| 25 | 1 | 2.749452 | 1.894654 | −2.243728 |
| 26 | 1 | 3.569664 | 2.252452 | −0.714563 |
| 27 | 1 | 1.803475 | 2.438366 | −0.840373 |
| 28 | 1 | −2.418889 | 2.500792 | −0.177894 |

B3LYP Energy = −690.35080320 a.u.

***Table S2.*** *Cont.*

| **Compound 1  Conformer 1c** | | **Standard Orientation (Ångstroms)** | | |
| --- | --- | --- | --- | --- |
| I | Atom | X | Y | Z |
| 1 | 6 | −2.210035 | −1.461491 | −0.783169 |
| 2 | 6 | −2.715789 | −0.433096 | −0.042734 |
| 3 | 8 | −1.704870 | 0.152964 | 0.680468 |
| 4 | 6 | −0.548170 | −0.515614 | 0.380502 |
| 5 | 6 | −0.805066 | −1.519520 | −0.505696 |
| 6 | 6 | −4.071187 | 0.171568 | 0.105324 |
| 7 | 6 | 0.696135 | −0.007281 | 1.046262 |
| 8 | 8 | 1.721777 | −0.975085 | 0.895482 |
| 9 | 6 | 2.857524 | −0.339429 | 0.271843 |
| 10 | 6 | 2.216163 | 0.854011 | −0.442894 |
| 11 | 8 | 1.165898 | 1.209947 | 0.473431 |
| 12 | 6 | 3.110578 | 2.057899 | −0.670947 |
| 13 | 6 | 3.575228 | −1.342338 | −0.612817 |
| 14 | 8 | −4.200053 | 1.444860 | −0.539208 |
| 15 | 1 | −2.773725 | −2.105349 | −1.444602 |
| 16 | 1 | −0.082807 | −2.218789 | −0.900616 |
| 17 | 1 | −4.333311 | 0.251988 | 1.170258 |
| 18 | 1 | −4.801744 | −0.486416 | −0.371395 |
| 19 | 1 | 0.501521 | 0.206043 | 2.106291 |
| 20 | 1 | 3.532445 | 0.029972 | 1.058696 |
| 21 | 1 | 1.775803 | 0.515010 | −1.393739 |
| 22 | 1 | 2.540371 | 2.881429 | −1.111816 |
| 23 | 1 | 3.922933 | 1.801231 | −1.359254 |
| 24 | 1 | 3.545557 | 2.401039 | 0.273634 |
| 25 | 1 | 3.917923 | −2.198693 | −0.023707 |
| 26 | 1 | 4.452730 | −0.877839 | −1.075857 |
| 27 | 1 | 2.912094 | −1.707025 | −1.404447 |
| 28 | 1 | −3.604097 | 2.057659 | −0.078879 |

B3LYP Energy = −690.35036078 a.u.

***Table S2.*** Cont.

| **Compound 1  Conformer 1d** | | **Standard Orientation (Ångstroms)** | | |
| --- | --- | --- | --- | --- |
| I | Atom | X | Y | Z |
| 1 | 6 | 2.176958 | 1.354125 | −0.912214 |
| 2 | 6 | 2.664443 | 0.167073 | −0.448630 |
| 3 | 8 | 1.682868 | −0.489909 | 0.253807 |
| 4 | 6 | 0.563910 | 0.298844 | 0.225656 |
| 5 | 6 | 0.813950 | 1.439955 | −0.477434 |
| 6 | 6 | 3.993164 | −0.507123 | −0.517340 |
| 7 | 6 | −0.645974 | −0.251483 | 0.920264 |
| 8 | 8 | −1.594840 | 0.790789 | 1.076419 |
| 9 | 6 | −2.840164 | 0.363045 | 0.485870 |
| 10 | 6 | −2.381311 | −0.702128 | −0.514768 |
| 11 | 8 | −1.273574 | −1.297624 | 0.183594 |
| 12 | 6 | −3.393231 | −1.779086 | −0.857737 |
| 13 | 6 | −3.560679 | 1.564477 | −0.098174 |
| 14 | 8 | 4.704315 | −0.493191 | 0.726506 |
| 15 | 1 | 2.726145 | 2.083428 | −1.492106 |
| 16 | 1 | 0.115038 | 2.244824 | −0.651578 |
| 17 | 1 | 4.620635 | 0.029390 | −1.233199 |
| 18 | 1 | 3.873959 | −1.538470 | −0.879822 |
| 19 | 1 | −0.364399 | −0.684017 | 1.890223 |
| 20 | 1 | −3.456148 | −0.112704 | 1.263924 |
| 21 | 1 | −2.017417 | −0.208996 | −1.429851 |
| 22 | 1 | −2.944712 | −2.532285 | −1.512906 |
| 23 | 1 | −4.250082 | −1.341363 | −1.381028 |
| 24 | 1 | −3.752107 | −2.275502 | 0.050021 |
| 25 | 1 | −2.956855 | 2.037937 | −0.879624 |
| 26 | 1 | −3.765390 | 2.305741 | 0.680572 |
| 27 | 1 | −4.518385 | 1.257011 | −0.532163 |
| 28 | 1 | 4.193175 | −1.031710 | 1.351988 |

B3LYP Energy = −690.35035463 a.u.

***Table S2.*** Cont.

| **Compound 1  Conformer 1e** | | **Standard Orientation (Ångstroms)** | | |
| --- | --- | --- | --- | --- |
| I | Atom | X | Y | Z |
| 1 | 6 | −2.468961 | −1.322676 | 0.945842 |
| 2 | 6 | −2.845548 | −0.404516 | 0.008858 |
| 3 | 8 | −1.731977 | 0.075237 | −0.637386 |
| 4 | 6 | −0.644249 | −0.547660 | −0.088609 |
| 5 | 6 | −1.041032 | −1.419689 | 0.880353 |
| 6 | 6 | −4.154247 | 0.177074 | −0.408207 |
| 7 | 6 | 0.689404 | −0.156820 | −0.633306 |
| 8 | 8 | 1.135948 | 1.056223 | −0.048318 |
| 9 | 6 | 2.569424 | 0.995616 | −0.119319 |
| 10 | 6 | 2.843921 | −0.489157 | 0.174039 |
| 11 | 8 | 1.656492 | −1.150065 | −0.318140 |
| 12 | 6 | 4.079206 | −1.068673 | −0.494563 |
| 13 | 6 | 3.168955 | 1.987081 | 0.859566 |
| 14 | 8 | −4.350065 | 1.522045 | 0.045193 |
| 15 | 1 | −3.132396 | −1.869678 | 1.601831 |
| 16 | 1 | −0.394258 | −2.056608 | 1.466078 |
| 17 | 1 | −4.957439 | −0.414446 | 0.037708 |
| 18 | 1 | −4.259783 | 0.120535 | −1.501255 |
| 19 | 1 | 0.632520 | −0.034389 | −1.728238 |
| 20 | 1 | 2.890319 | 1.228215 | −1.148081 |
| 21 | 1 | 2.892539 | −0.644057 | 1.261207 |
| 22 | 1 | 4.160786 | −2.140861 | −0.290259 |
| 23 | 1 | 4.982807 | −0.581909 | −0.110121 |
| 24 | 1 | 4.038111 | −0.923656 | −1.579487 |
| 25 | 1 | 2.868809 | 3.008067 | 0.604400 |
| 26 | 1 | 4.262495 | 1.936599 | 0.828292 |
| 27 | 1 | 2.837092 | 1.769039 | 1.880124 |
| 28 | 1 | −3.686021 | 2.072154 | −0.400923 |

B3LYP Energy = −690.34944135 a.u.

***Table S2.*** Cont.

| **Compound 1  Conformer 1f** | | **Standard Orientation (Ångstroms)** | | |
| --- | --- | --- | --- | --- |
| I | Atom | X | Y | Z |
| 1 | 6 | −2.446996 | −1.052582 | 1.216244 |
| 2 | 6 | −2.816424 | −0.025638 | 0.396786 |
| 3 | 8 | −1.722737 | 0.415598 | −0.308290 |
| 4 | 6 | −0.654637 | −0.346088 | 0.081109 |
| 5 | 6 | −1.043769 | −1.259568 | 1.014231 |
| 6 | 6 | −4.111367 | 0.653950 | 0.103192 |
| 7 | 6 | 0.657351 | −0.024940 | −0.554178 |
| 8 | 8 | 1.261095 | 1.093917 | 0.075846 |
| 9 | 6 | 2.672070 | 0.918285 | −0.128902 |
| 10 | 6 | 2.833152 | −0.603602 | 0.025540 |
| 11 | 8 | 1.553263 | −1.118579 | −0.404940 |
| 12 | 6 | 3.949470 | −1.228004 | −0.794649 |
| 13 | 6 | 3.439035 | 1.770788 | 0.864037 |
| 14 | 8 | −4.652089 | 0.319715 | −1.180751 |
| 15 | 1 | −3.099715 | −1.599749 | 1.882717 |
| 16 | 1 | −0.408447 | −1.992601 | 1.489724 |
| 17 | 1 | −3.994999 | 1.743163 | 0.199453 |
| 18 | 1 | −4.850690 | 0.329423 | 0.839434 |
| 19 | 1 | 0.518287 | 0.191657 | −1.626964 |
| 20 | 1 | 2.926108 | 1.207767 | −1.161908 |
| 21 | 1 | 2.958715 | −0.851820 | 1.088977 |
| 22 | 1 | 3.949945 | −2.316429 | −0.679192 |
| 23 | 1 | 4.923022 | −0.852424 | −0.459438 |
| 24 | 1 | 3.829865 | −0.991231 | −1.857360 |
| 25 | 1 | 3.214177 | 2.831224 | 0.713866 |
| 26 | 1 | 4.517030 | 1.630012 | 0.731697 |
| 27 | 1 | 3.173500 | 1.498303 | 1.890930 |
| 28 | 1 | −4.040358 | 0.671893 | −1.847320 |

B3LYP Energy = −690.34946389 a.u.


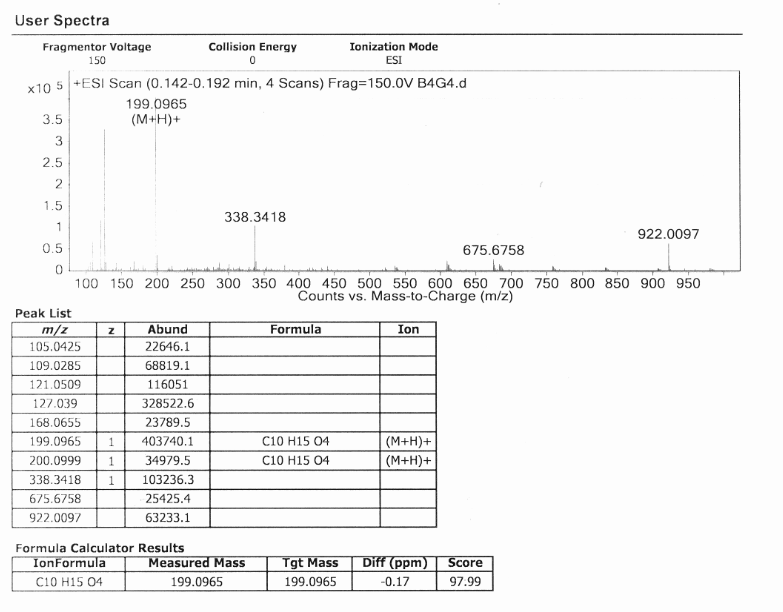


**Figure S1.** HRESIMS spectrum of compound **1**.


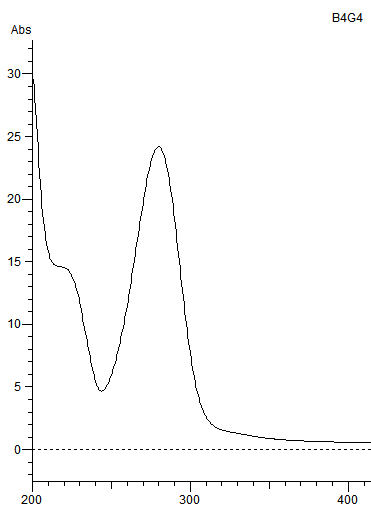


**Figure S2.** UV spectrum of compound **1** in MeOH.


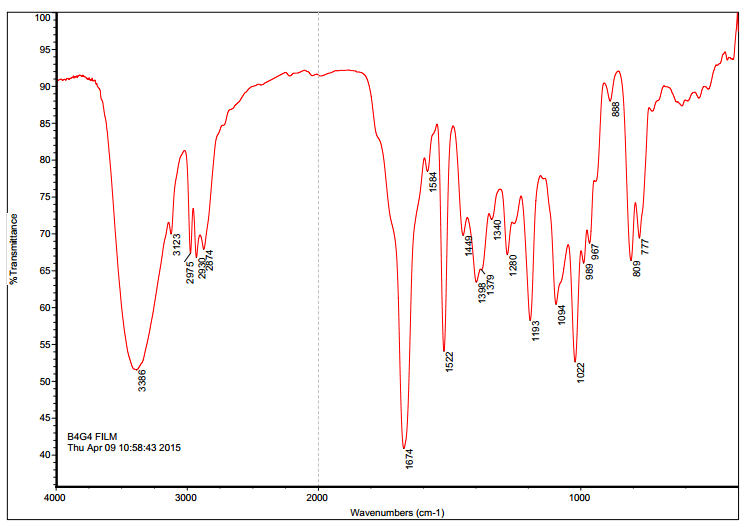


**Figure S3.** IR spectrum of compound **1**.

|  | 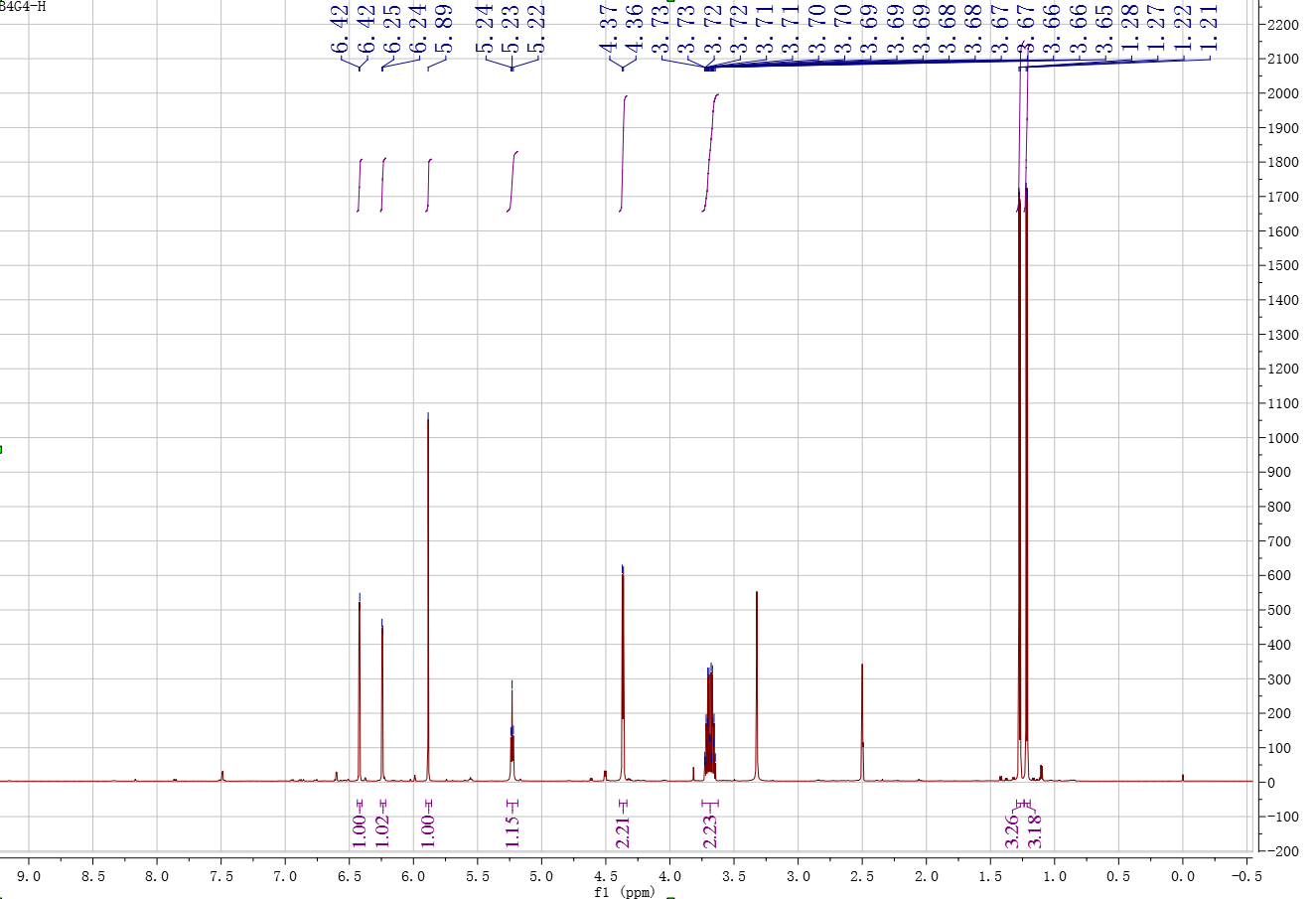 |
| --- | --- |
| (**a**) | (**b**) |

**Figure S4.** (**a**) The structure of compound **1**; (**b**) ^1^H NMR spectrum of compound **1** in DMSO-*d*_6_.


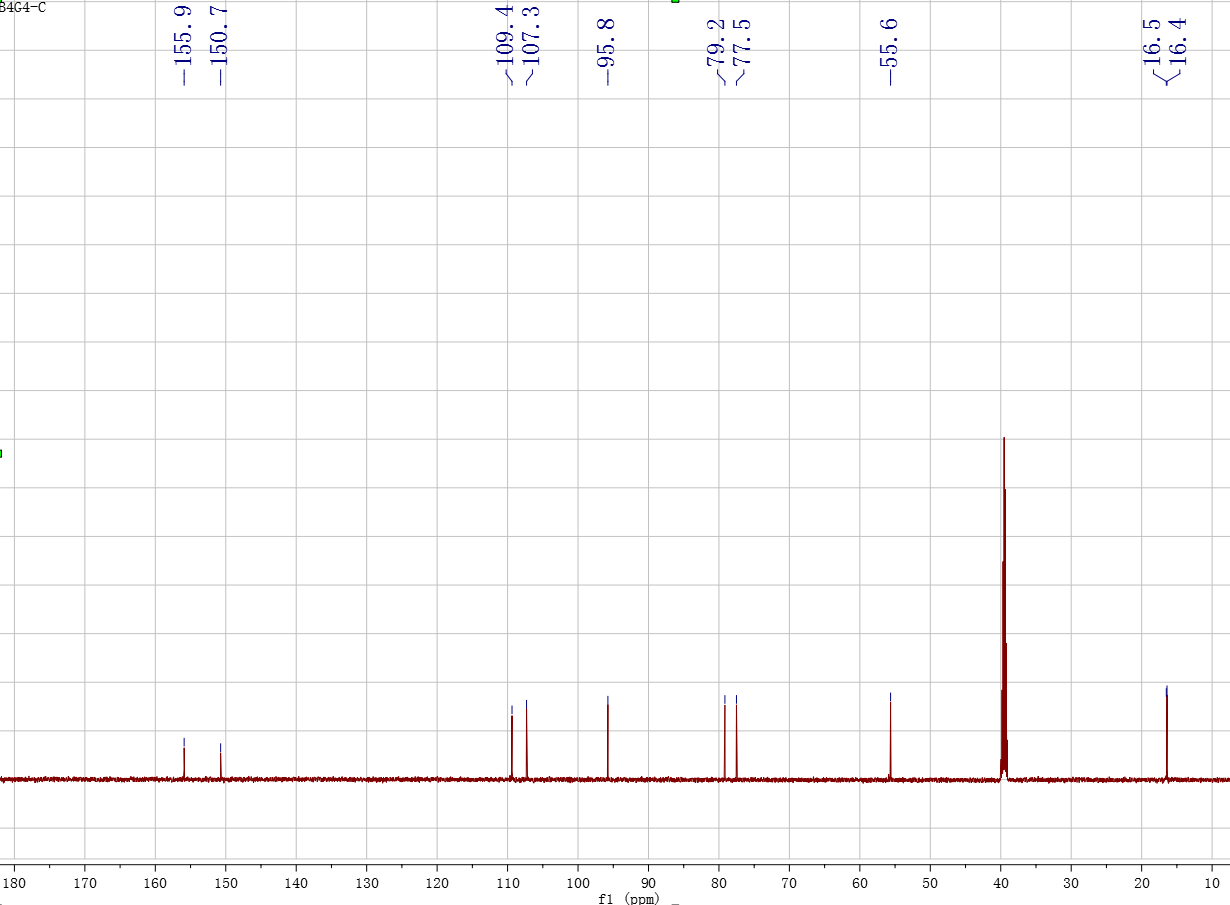


**Figure S5.** ^13^C NMR spectrum of compound **1** in DMSO-*d*_6_.


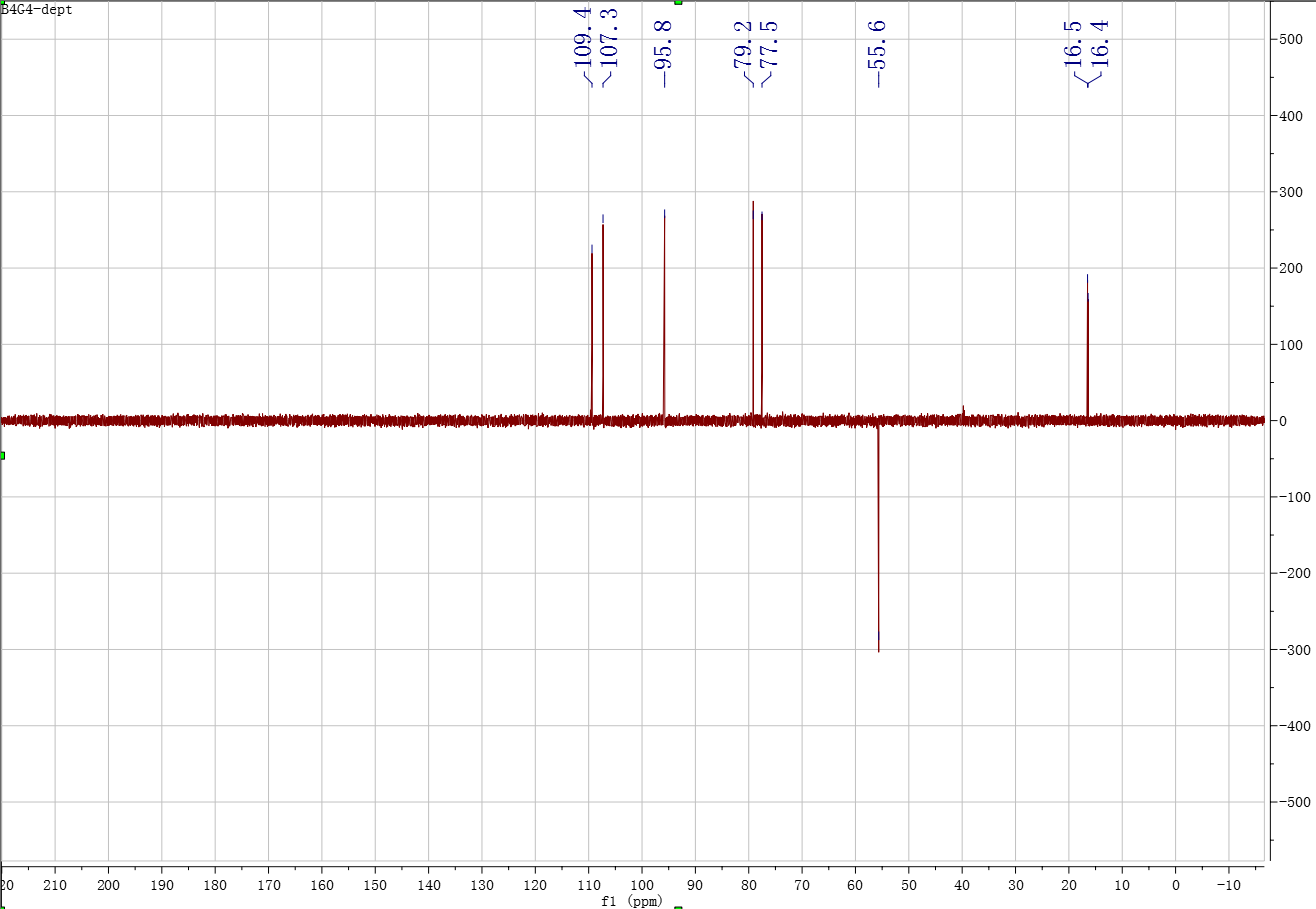


**Figure S6.** DEPT spectrum of compound **1** in DMSO-*d*_6_.


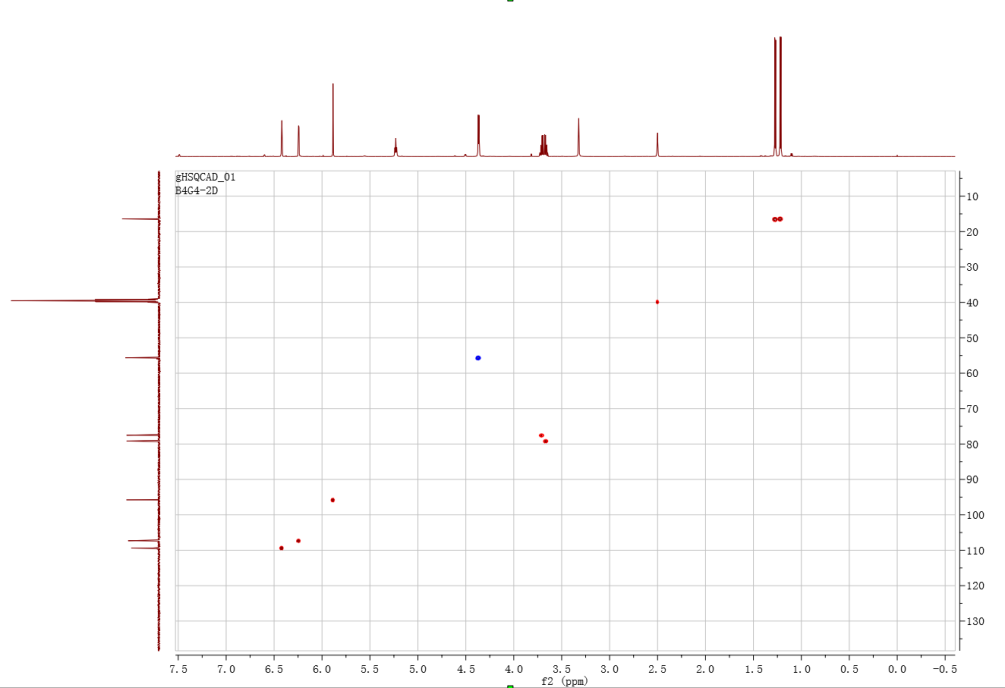


**Figure S7.** HSQC spectrum of compound **1** in DMSO-*d*_6_.


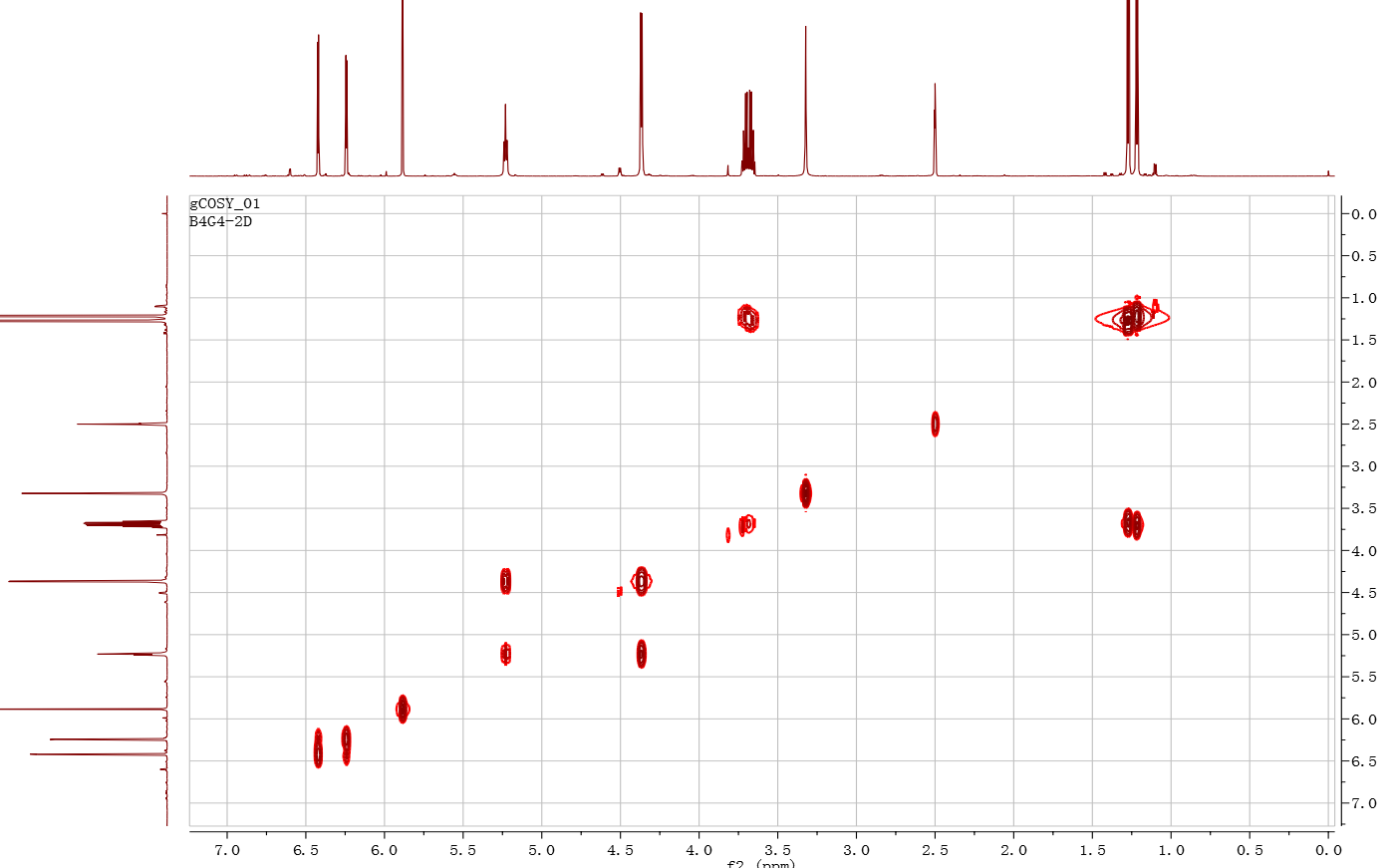


**Figure S8.** ^1^H-^1^H COSY spectrum of compound **1** in DMSO-*d*_6_.


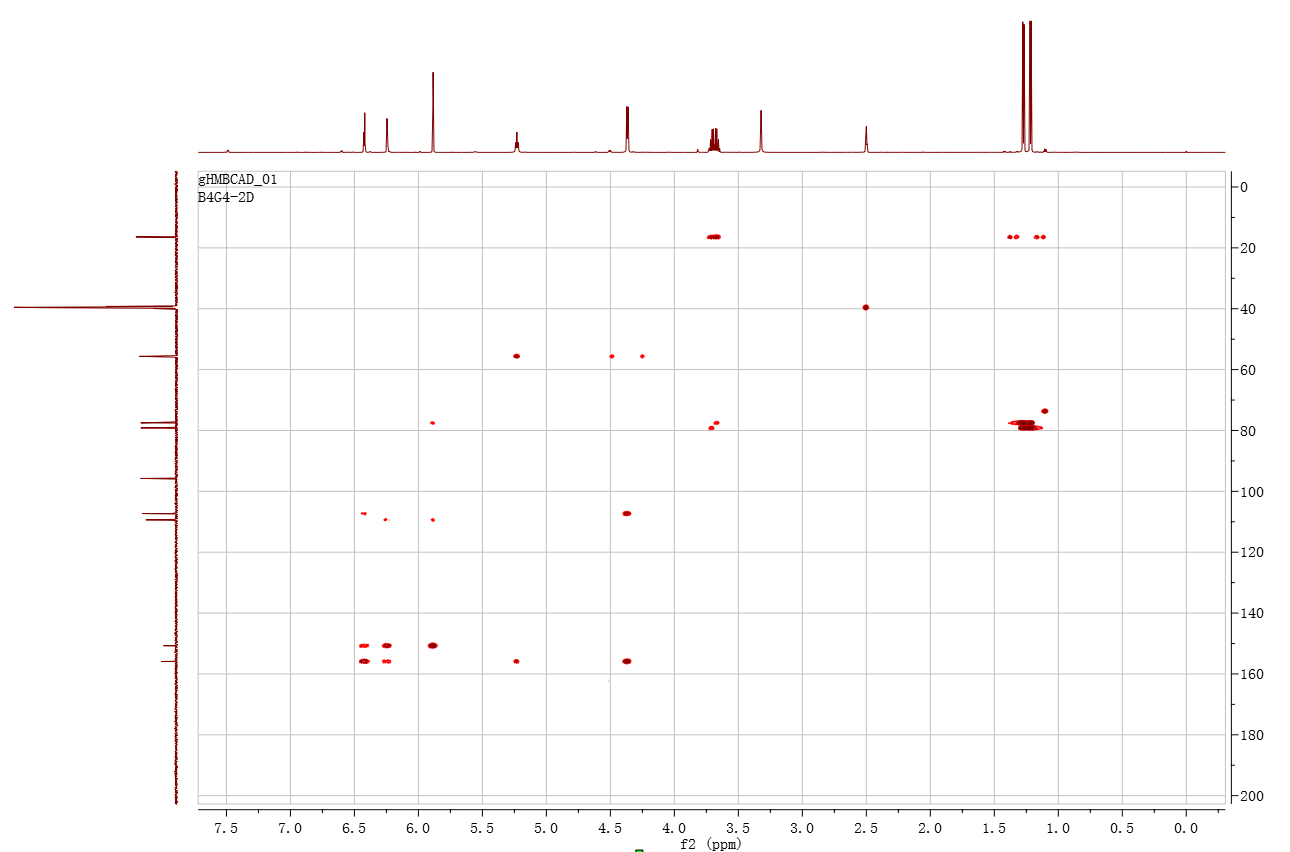


**Figure S9.** HMBC spectrum of compound **1** in DMSO-*d*_6_.


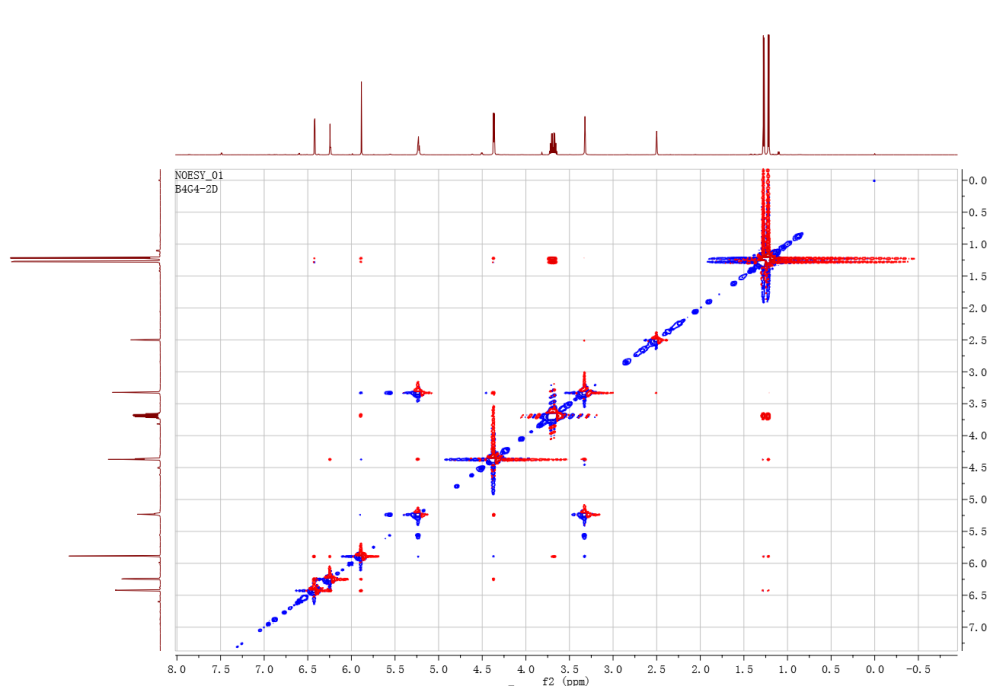


**Figure S10.** NOESY spectrum of compound **1** in DMSO-*d*_6_.


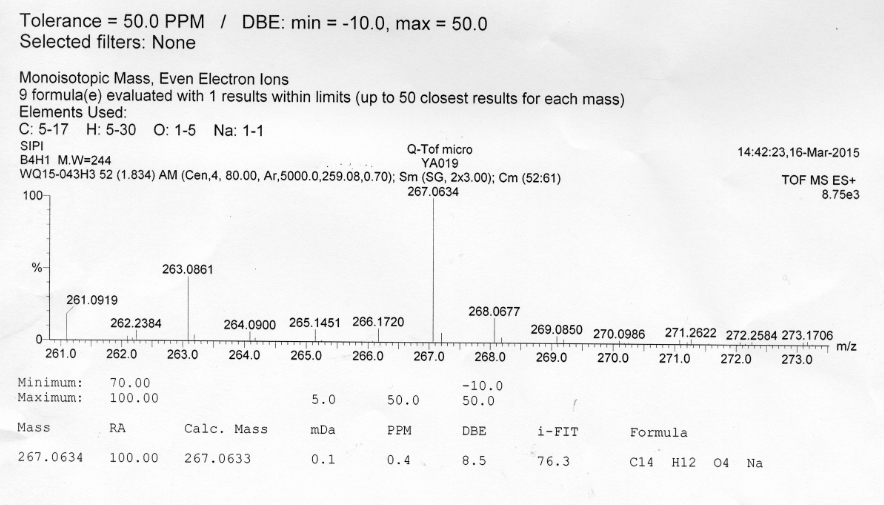


**Figure S11.** HRESIMS spectrum of compound **2**.


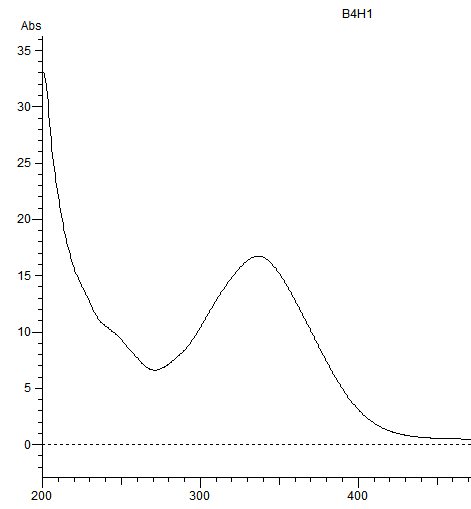


**Figure S12.** UV spectrum of compound **2** in MeOH.


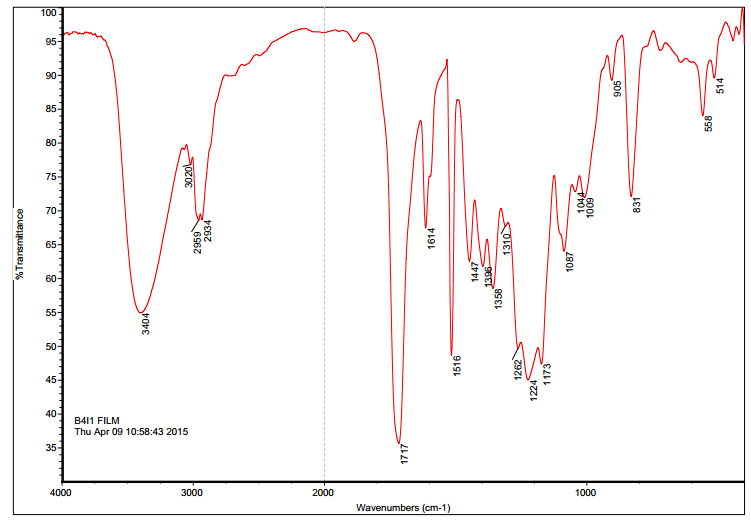


**Figure S13.** IR spectrum of compound **2**.

|  | 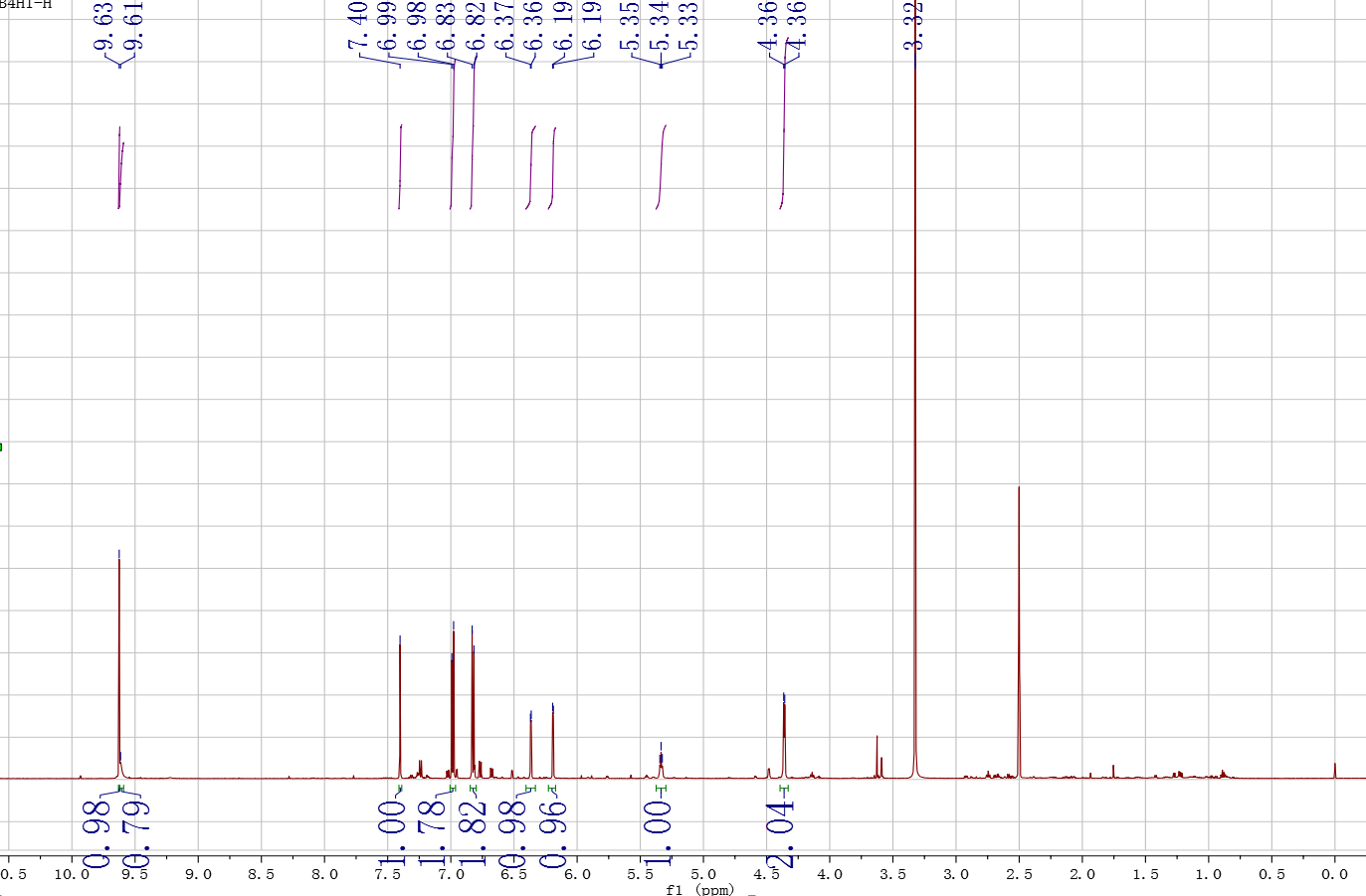 |
| --- | --- |
| (**a**) | (**b**) |

**Figure S14.** (**a**) The structure of compound **2**; (**b**) ^1^H NMR spectrum of compound **2** in DMSO-*d*_6_.


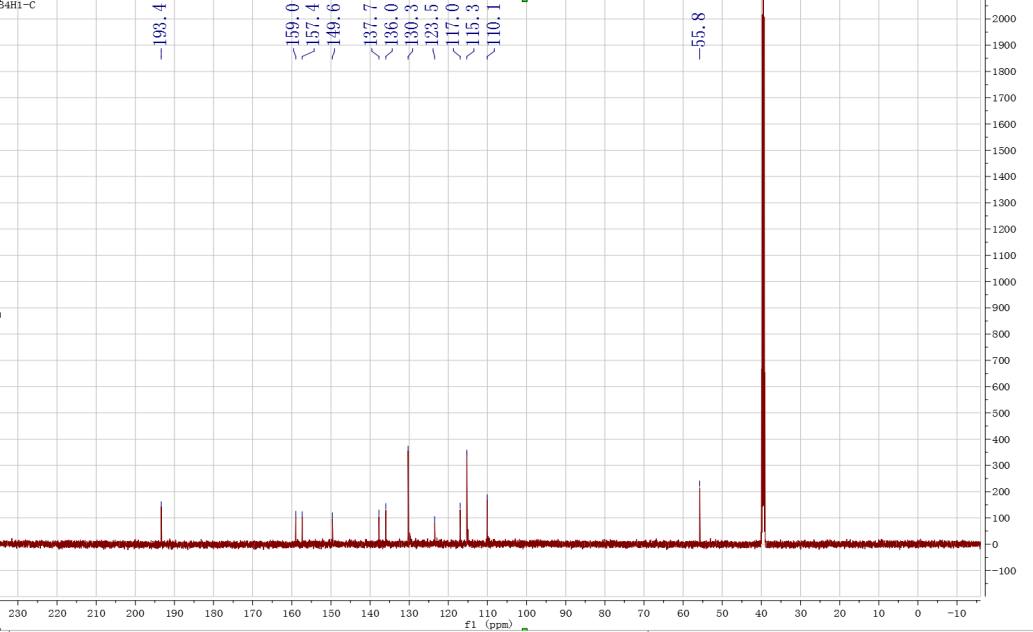


**Figure S15.** ^13^C NMR spectrum of compound **2** in DMSO-*d*_6_.


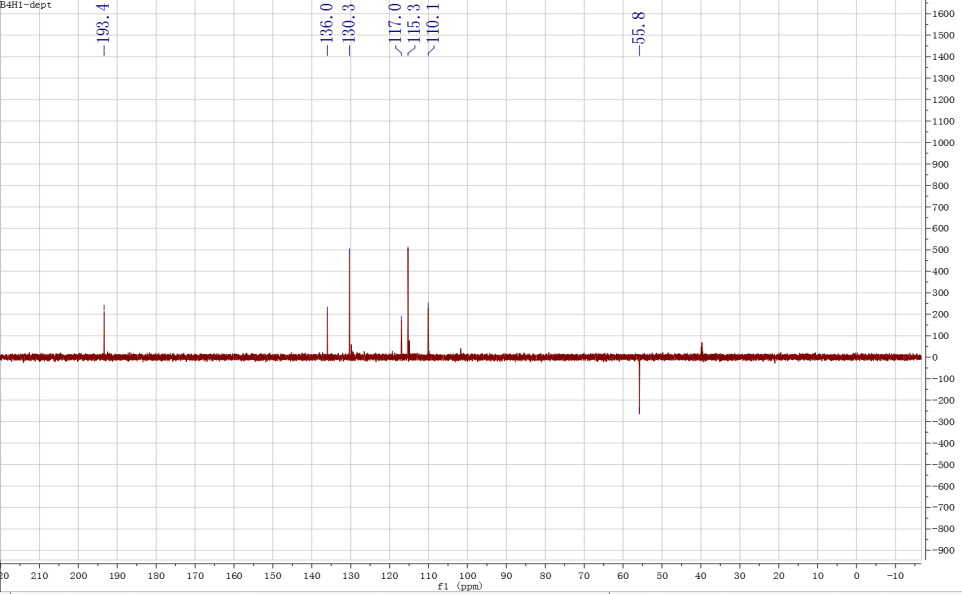


**Figure S16.** DEPT spectrum of compound **2** in DMSO-*d*_6_.


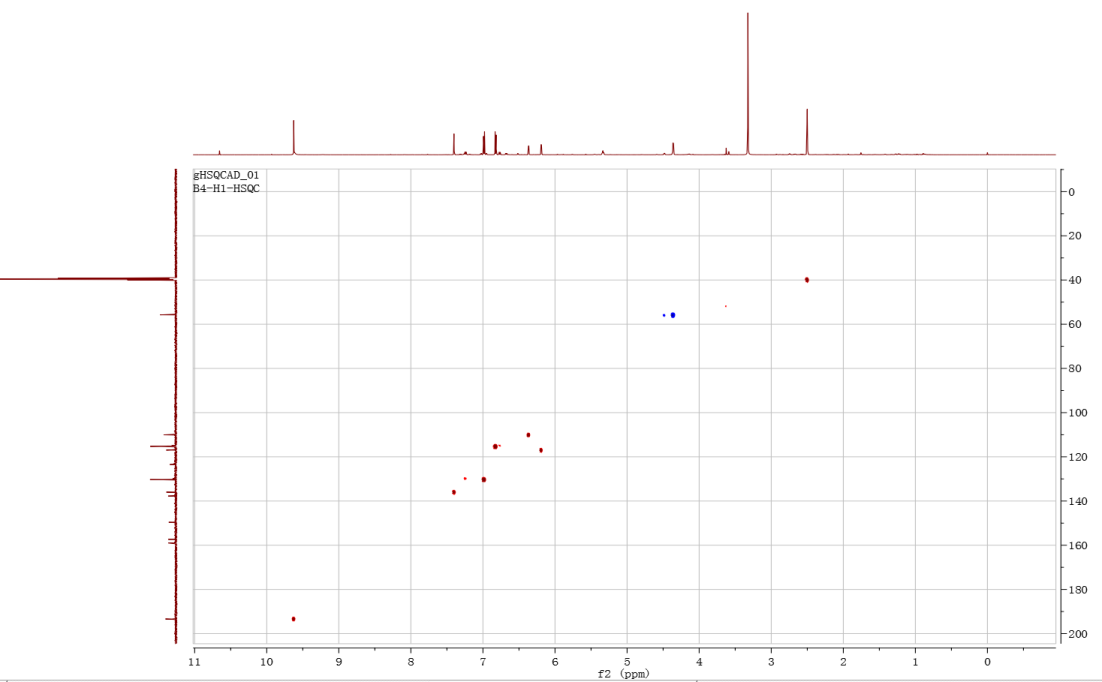


**Figure S17.** HSQC spectrum of compound **2** in DMSO-*d*_6_.


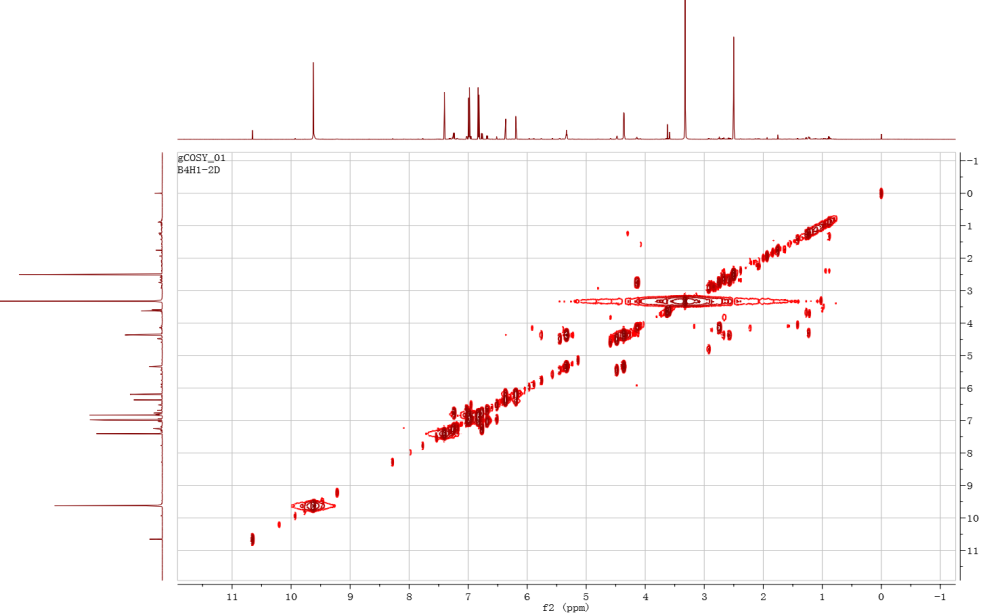


**Figure S18.** ^1^H-^1^H COSY spectrum of compound **2** in DMSO-*d*_6_.


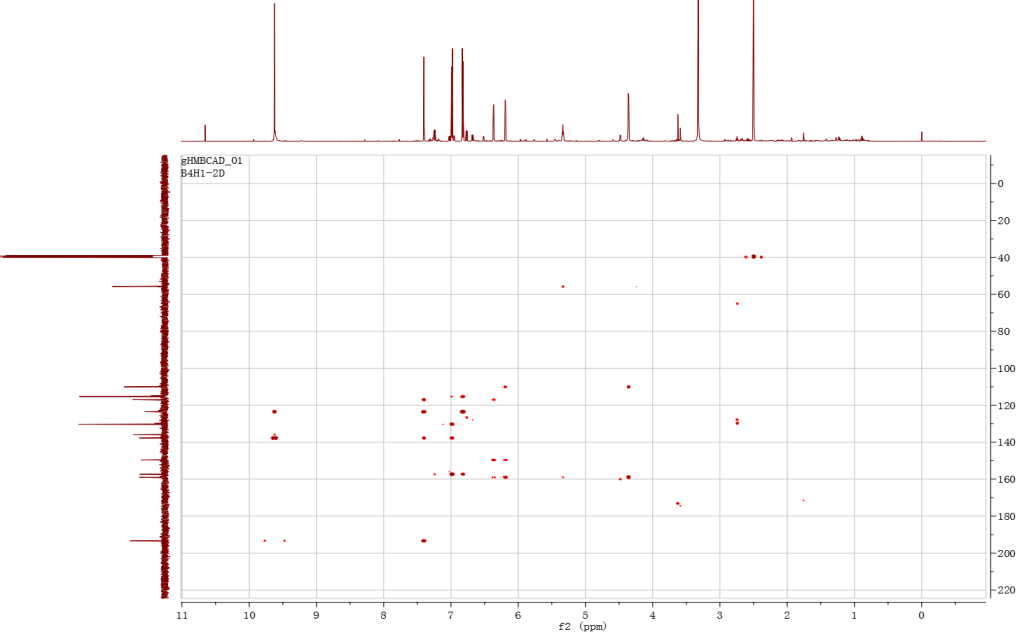


**Figure S19.** HMBC spectrum of compound **2** in DMSO-*d*_6_.


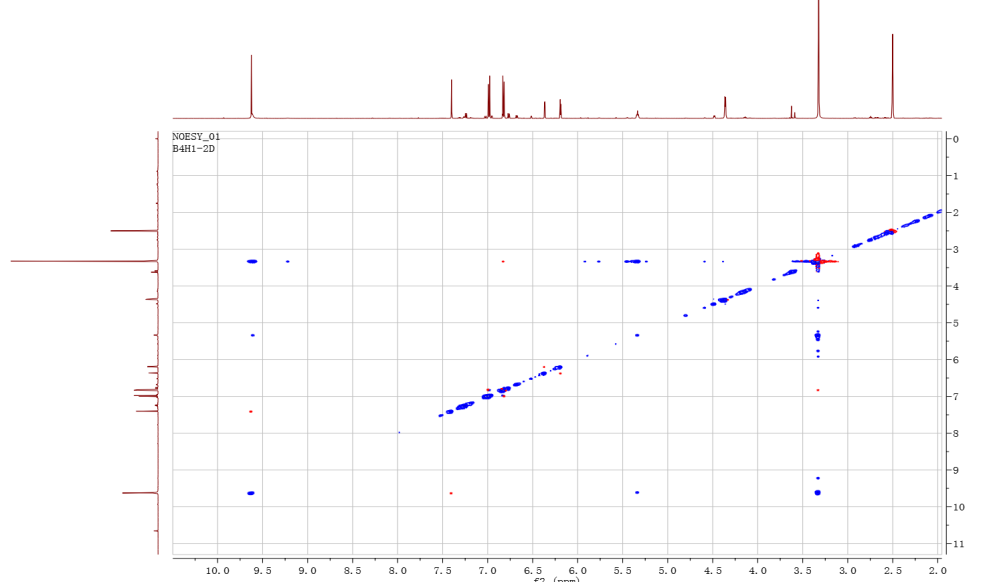


**Figure S20.** NOESY spectrum of compound **2** in DMSO-*d*_6_.


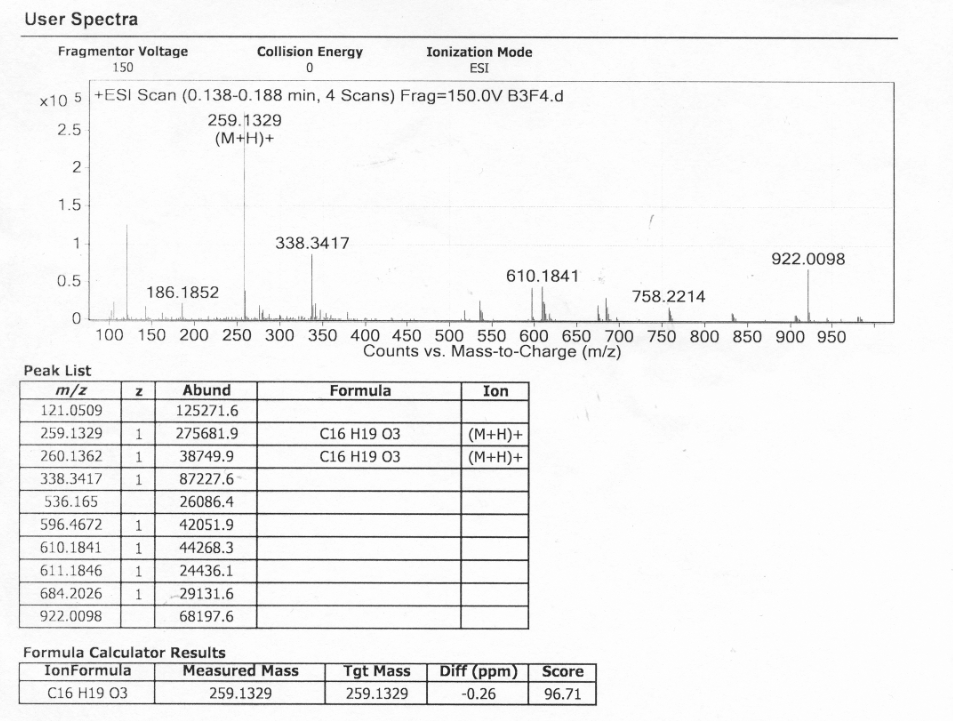


**Figure S21.** HRESIMS spectrum of compound **3**.


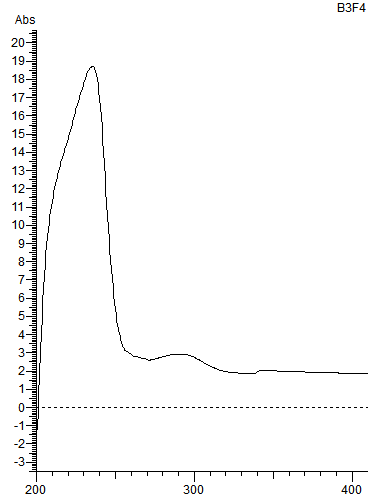


**Figure S22.** UV spectrum of compound **3** in MeOH.


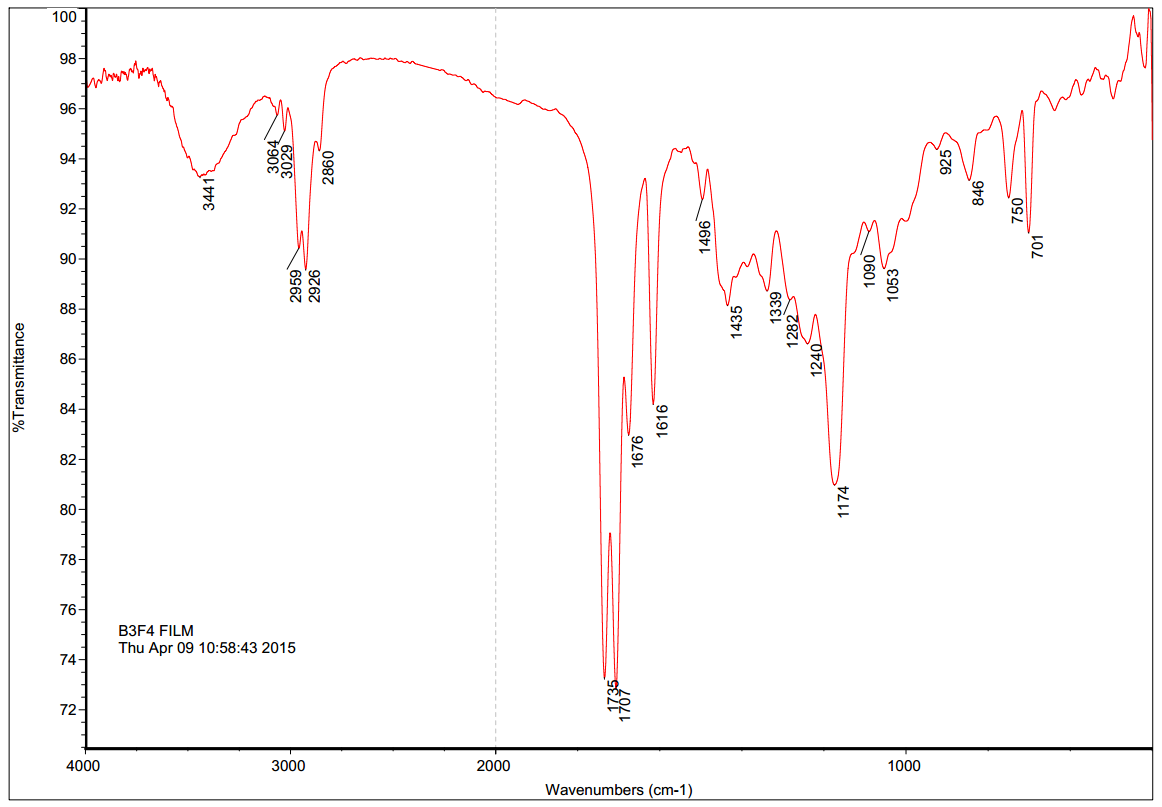


**Figure S23.** IR spectrum of compound **3**.

|  |
| --- |
| (**a**) |
| 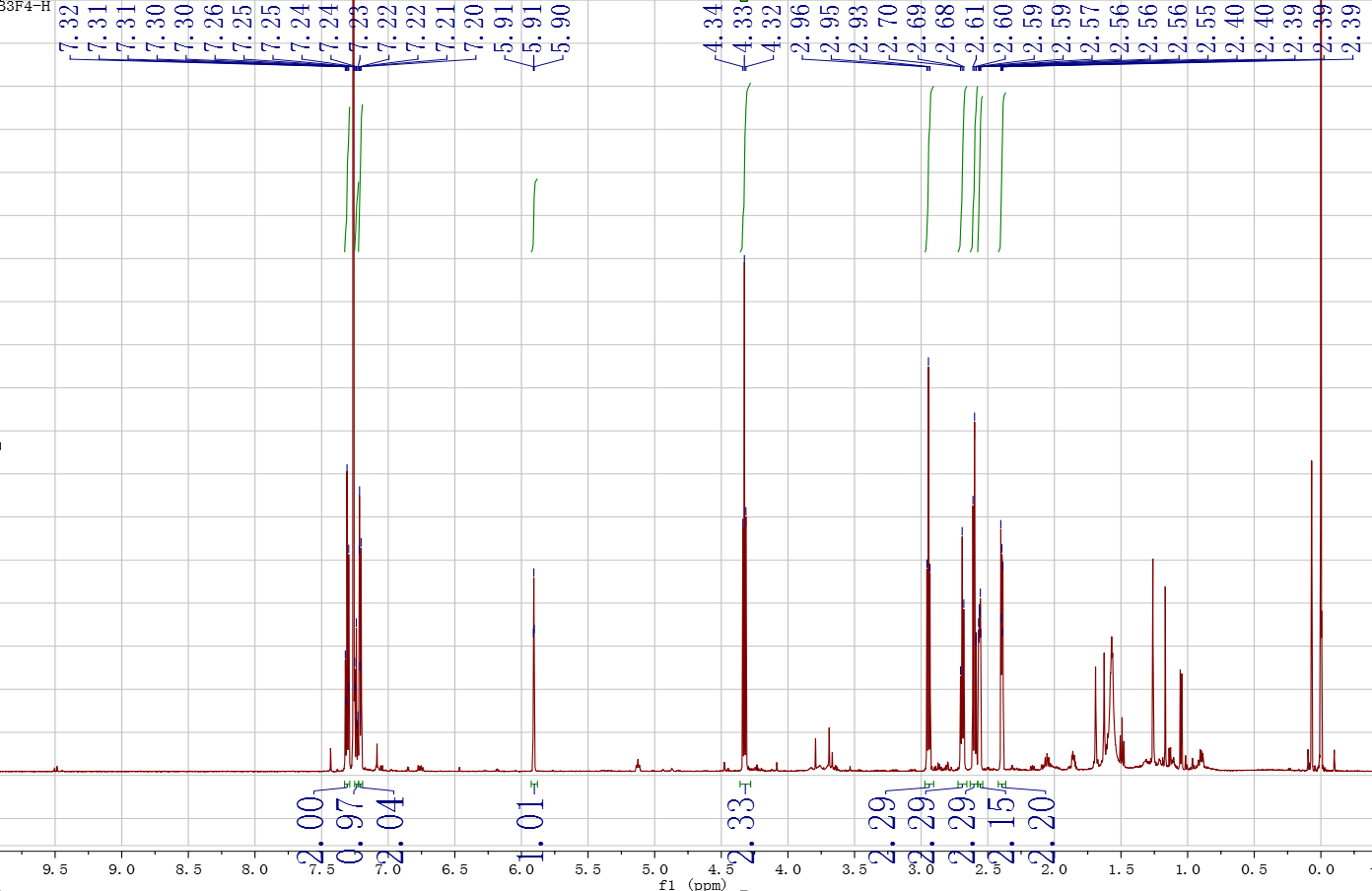 |
| (**b**) |

**Figure S24.** (**a**) The structure of compound **3**; (**b**) ^1^H NMR spectrum of compound **3** in DMSO-*d*_6_.


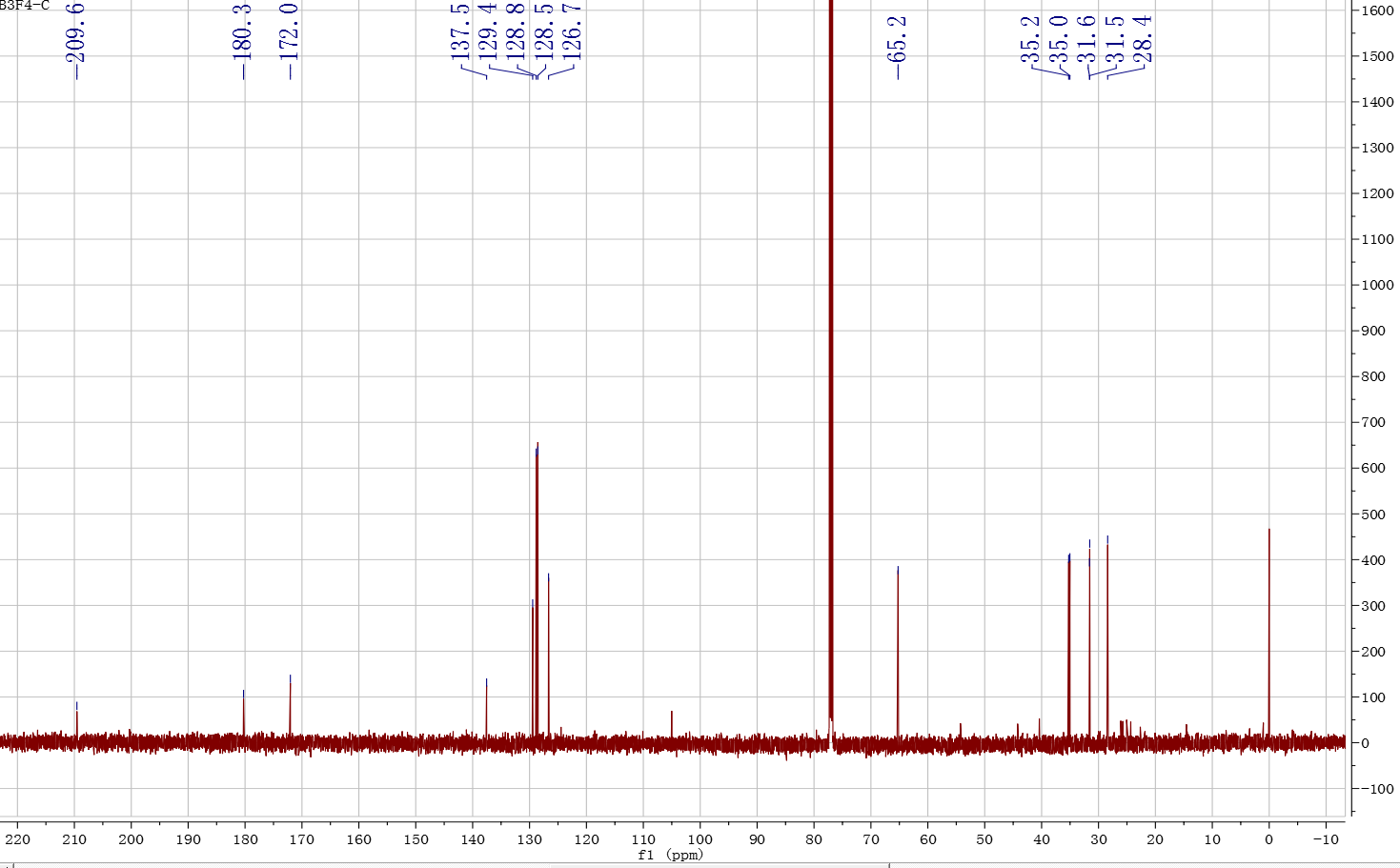


**Figure S25.** ^13^C NMR spectrum of compound **3** in DMSO-*d*_6_.


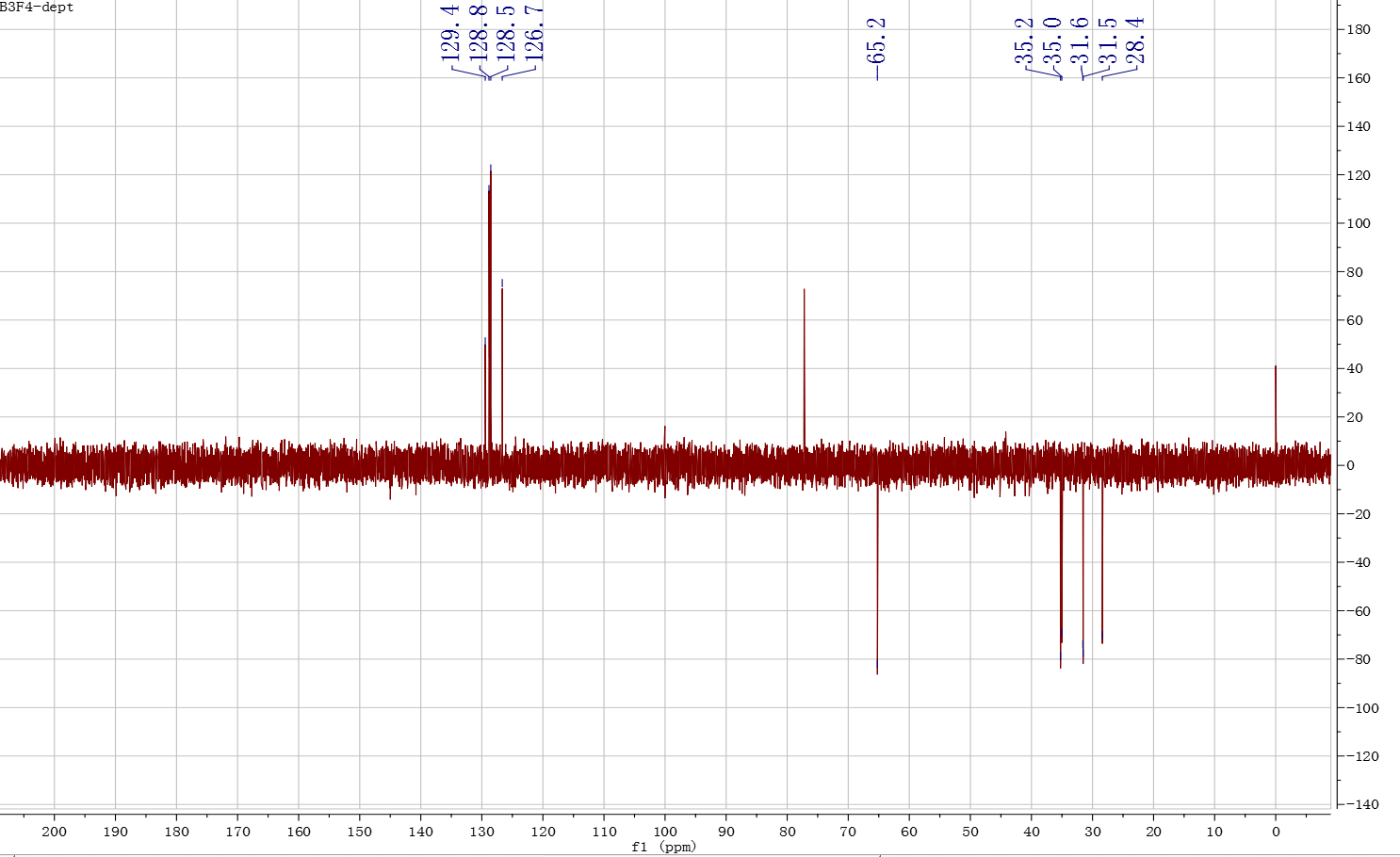


**Figure S26.** DEPT spectrum of compound **3** in DMSO-*d*_6_.


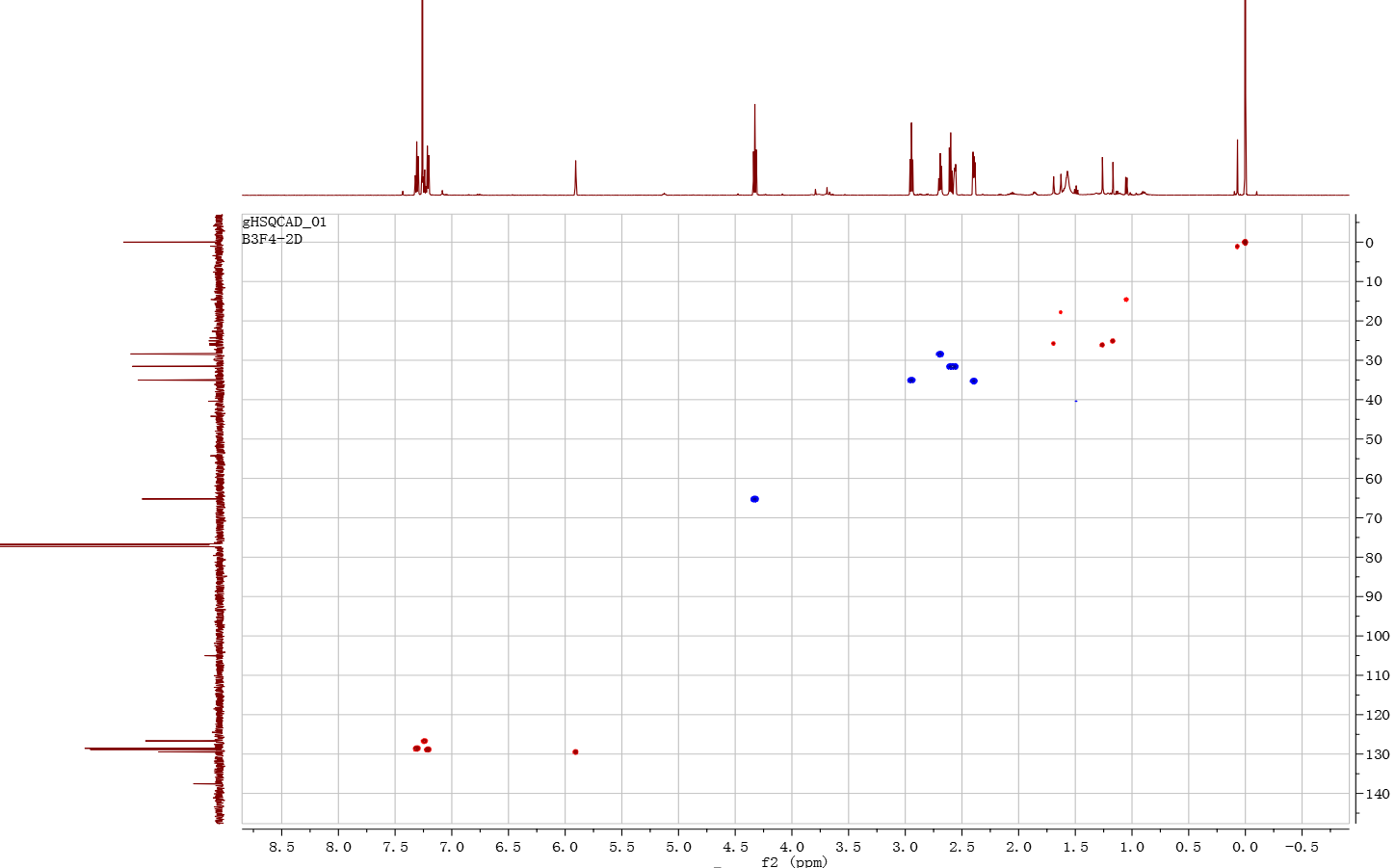


**Figure S27.** HSQC spectrum of compound **3** in DMSO-*d*_6_.


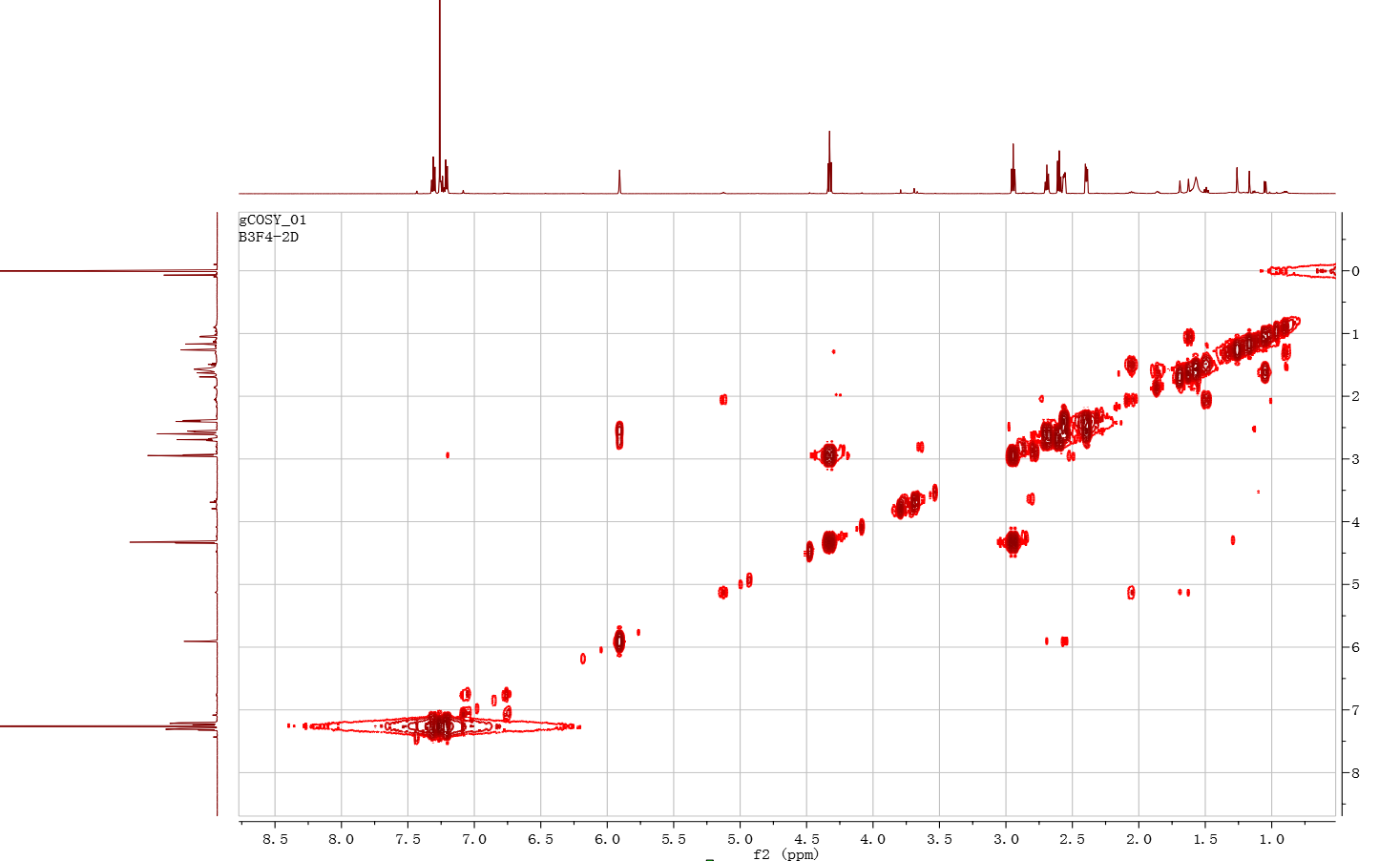


**Figure S28.** ^1^H-^1^H COSY spectrum of compound **3** in DMSO-*d*_6_.


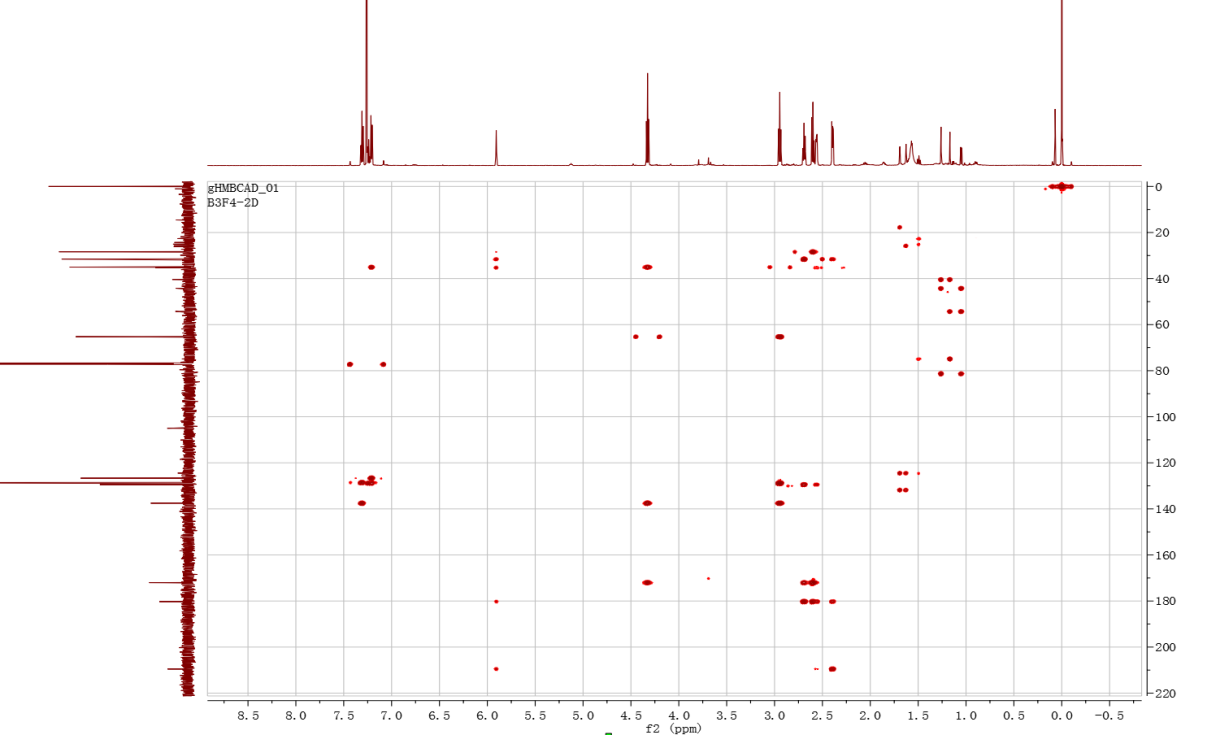


**Figure S29.** HMBC spectrum of compound **3** in DMSO-*d*_6_.


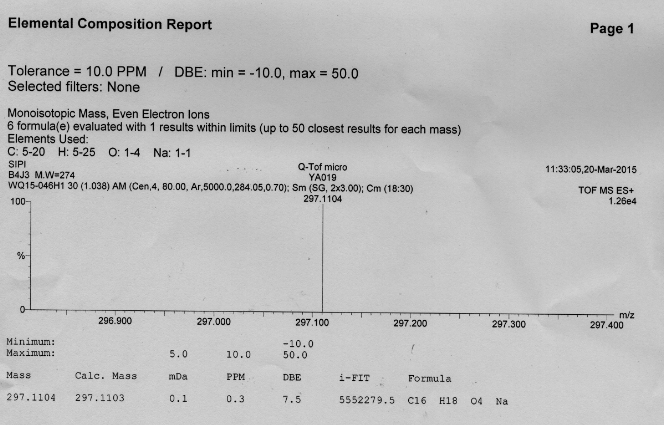


**Figure S30.** HRESIMS spectrum of compound **4**.


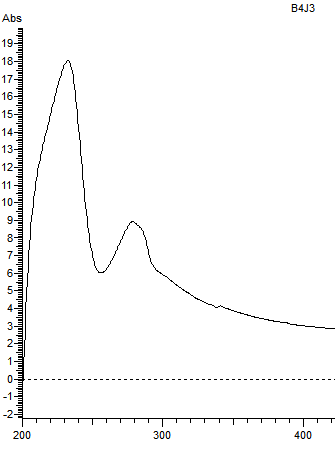


**Figure S31.** UV spectrum of compound **4** in MeOH.


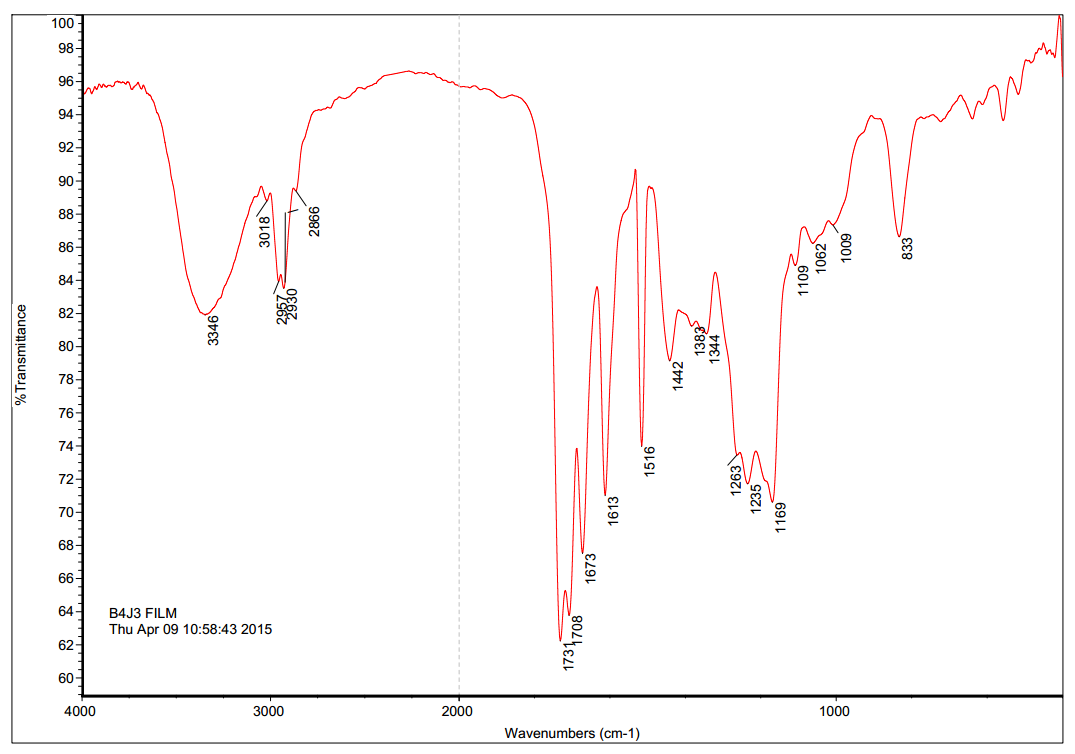


**Figure S32.** IR spectrum of compound **4**.

|  |
| --- |
| (**a**) |
| 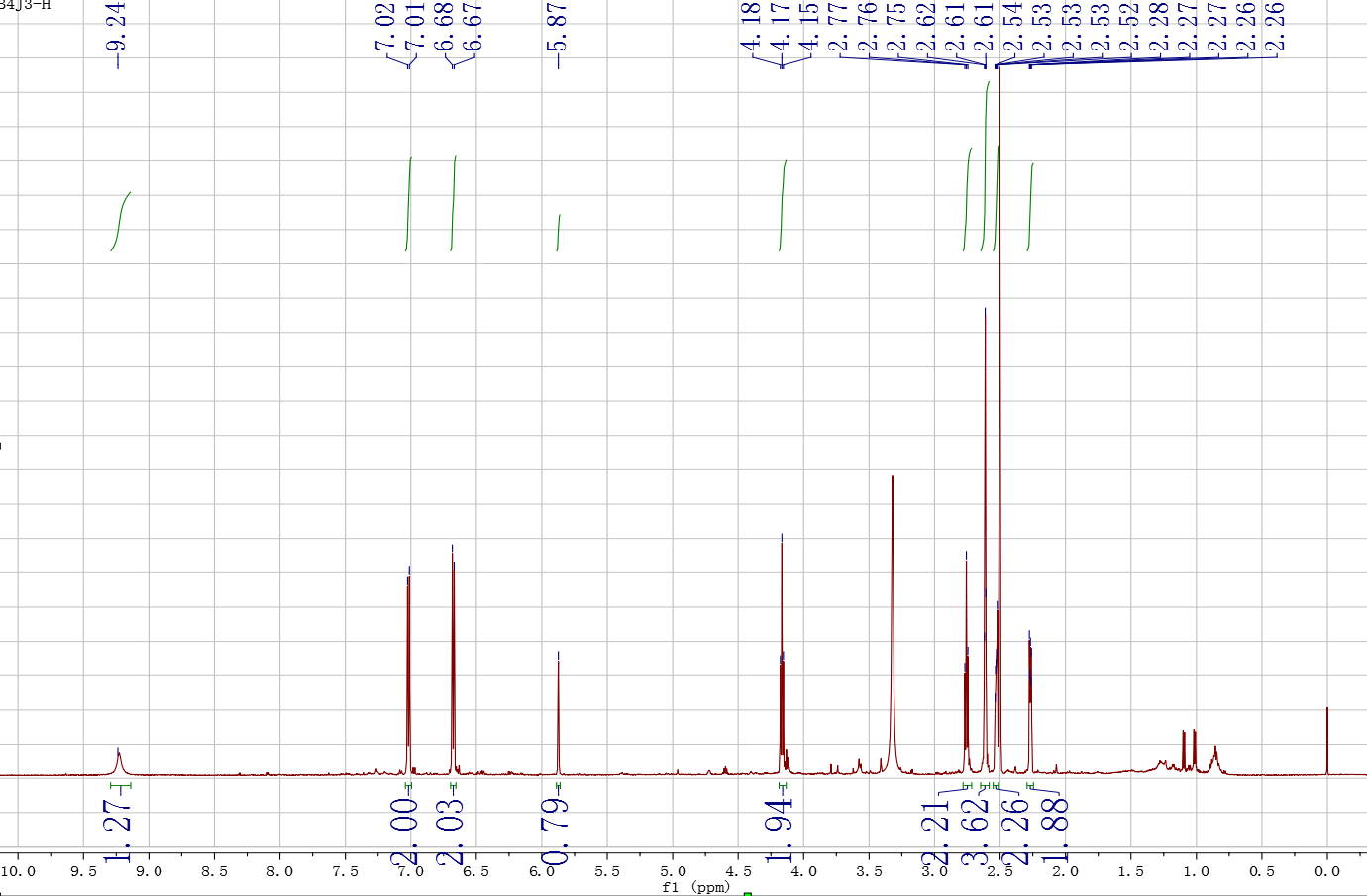 |
| (**b**) |

**Figure S33.** (**a**) The structure of compound **4**; (**b**) ^1^H NMR spectrum of compound **4** in DMSO-*d*_6_.


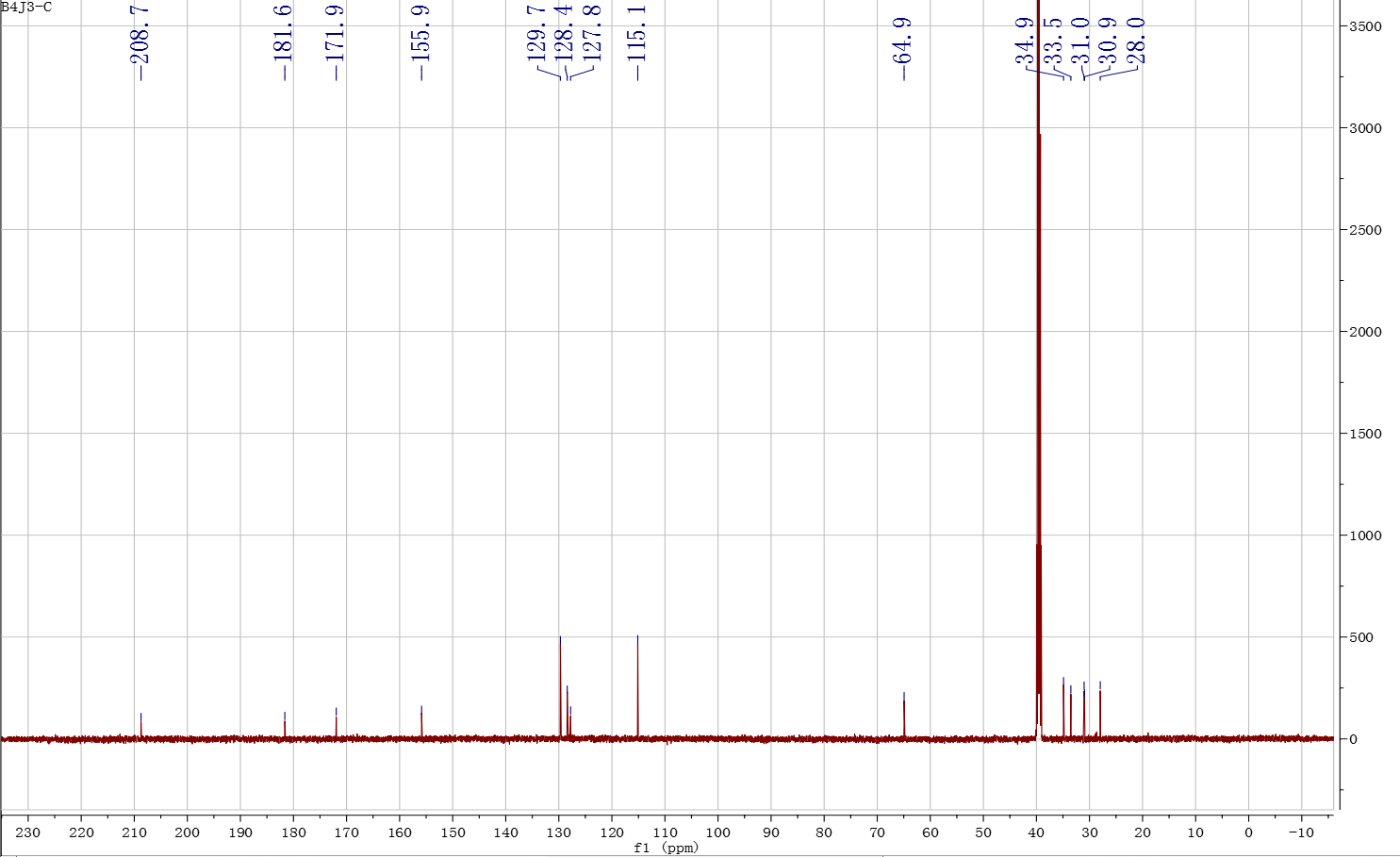


**Figure S34.** ^13^C NMR spectrum of compound **4** in DMSO-*d*_6_


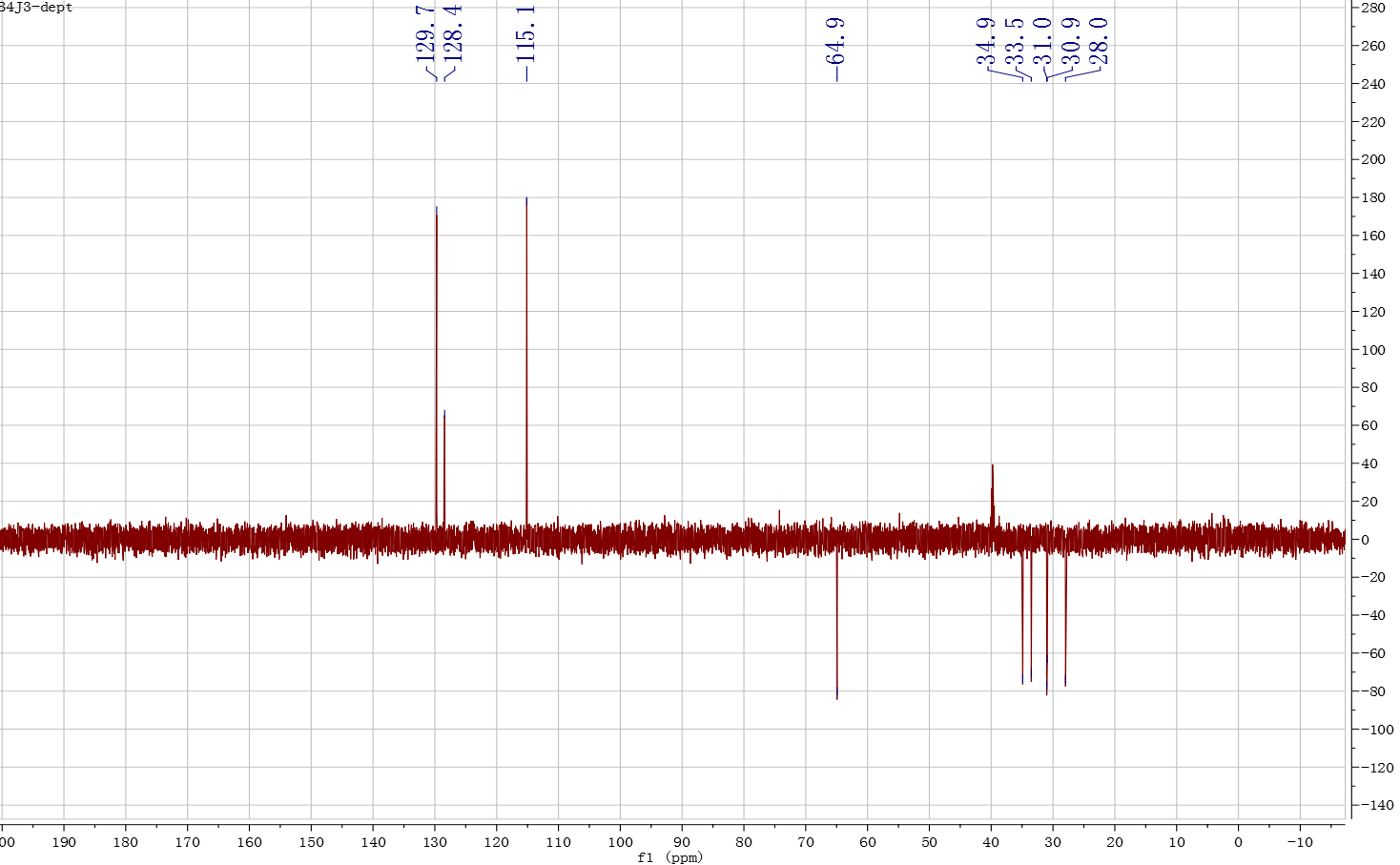


**Figure S35.** DEPT spectrum of compound **4** in DMSO-*d*_6_.


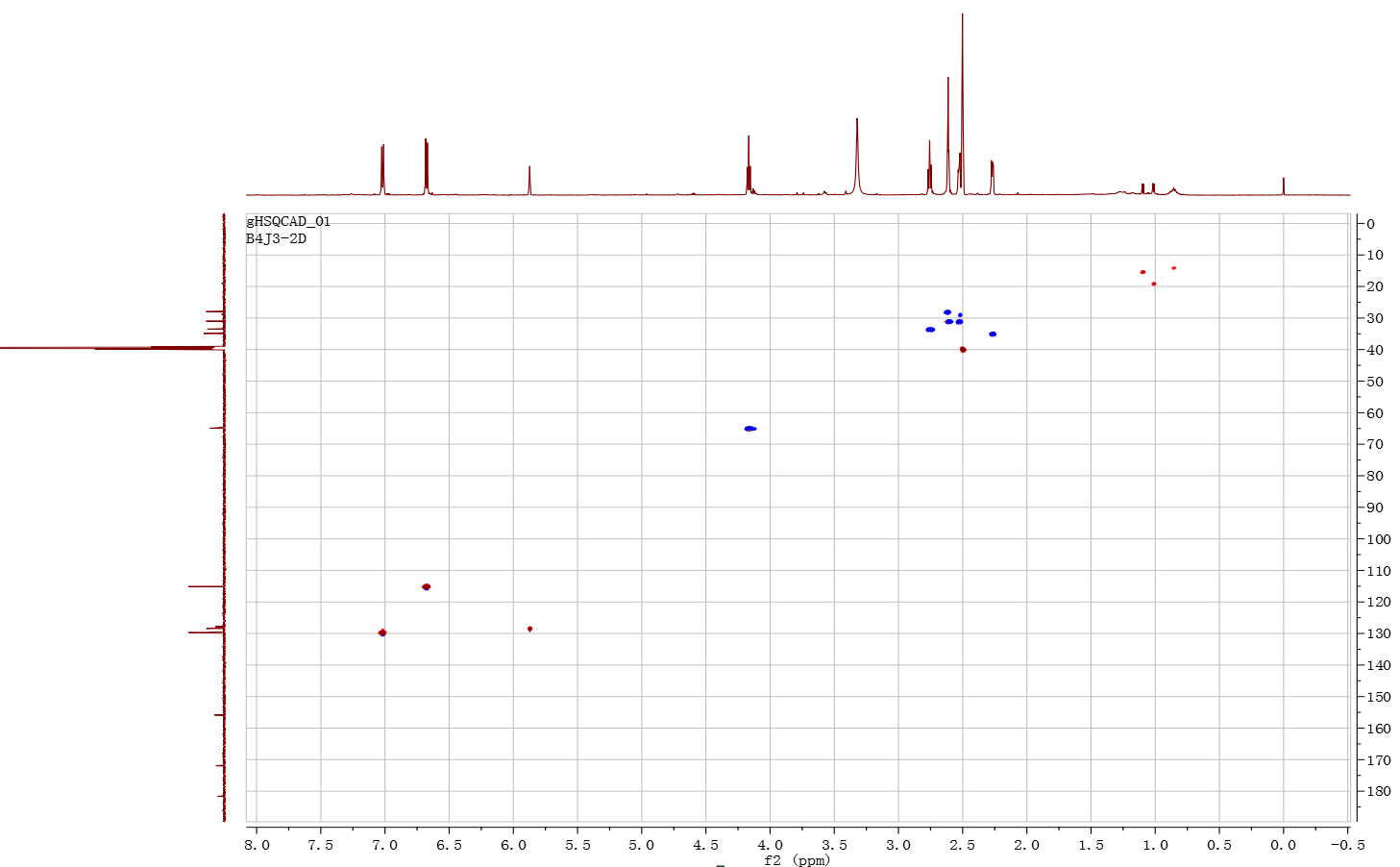


**Figure S36.** HSQC spectrum of compound **4** in DMSO-*d*_6_.


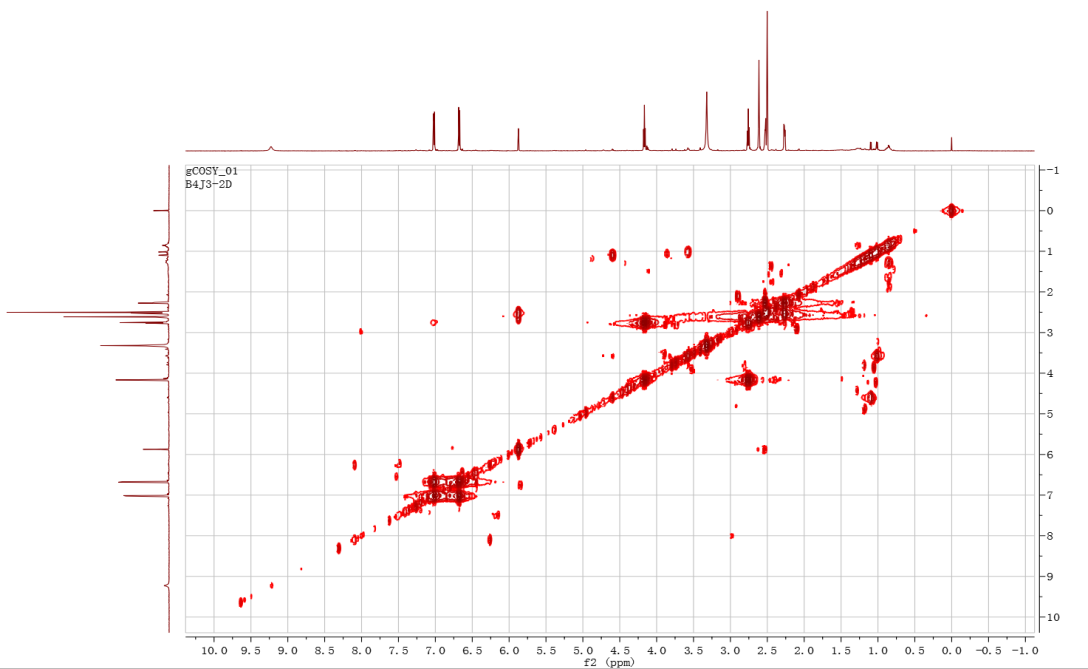


**Figure S37.** ^1^H-^1^H COSY spectrum of compound **4** in DMSO-*d*_6_.


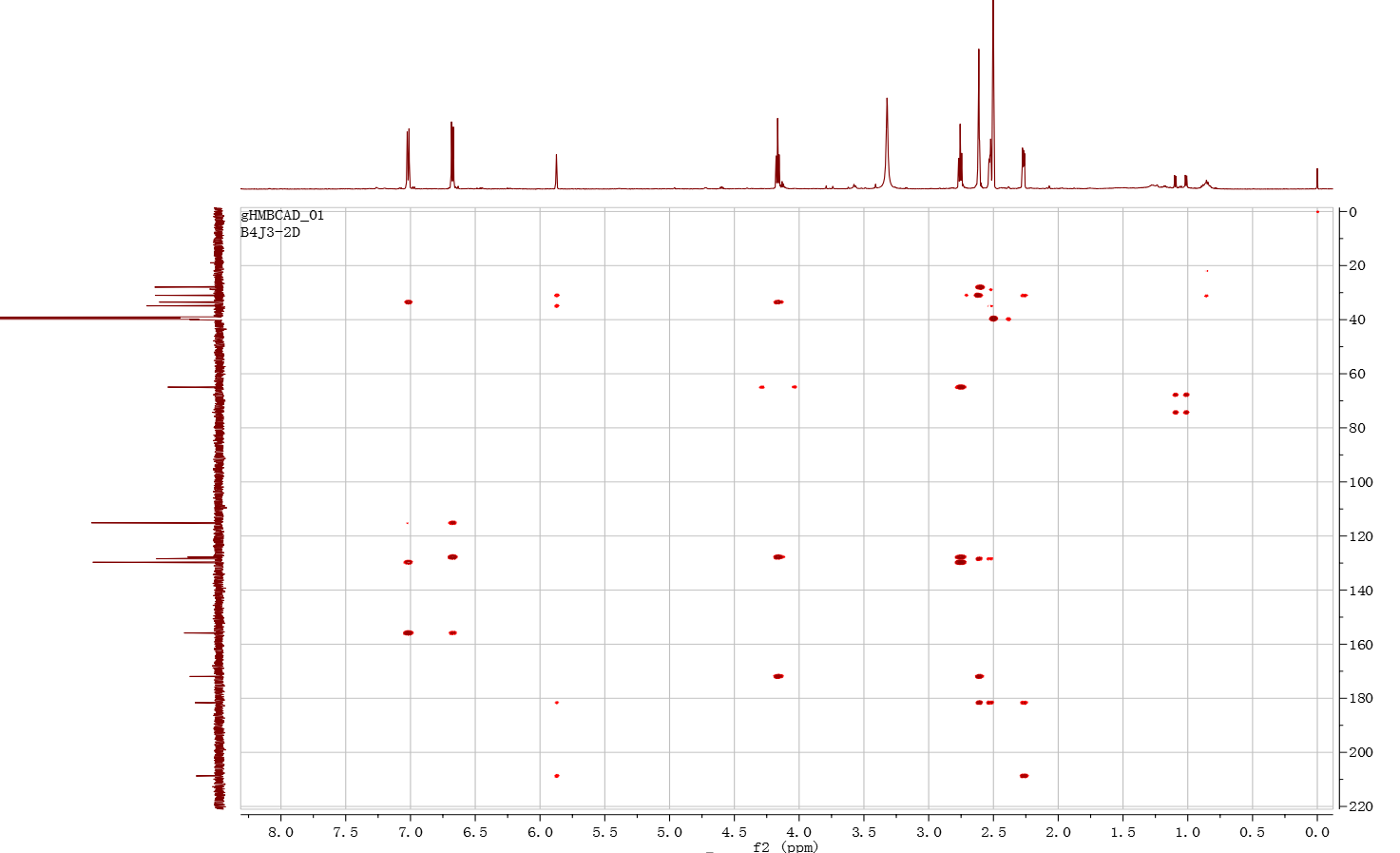


**Figure S38.** HMBC spectrum of compound **4** in DMSO-*d*_6_.


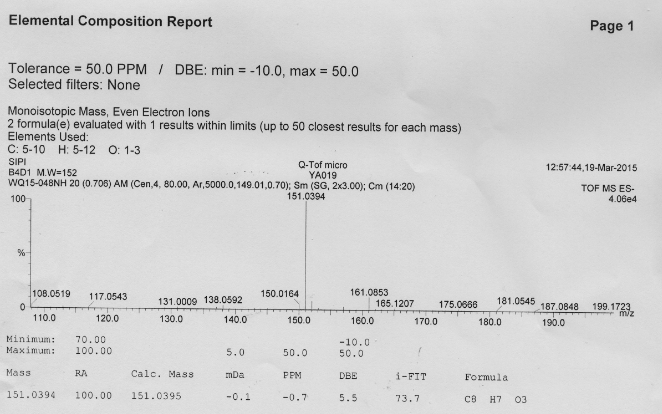


**Figure S39.** HRESIMS spectrum of compound **5**.


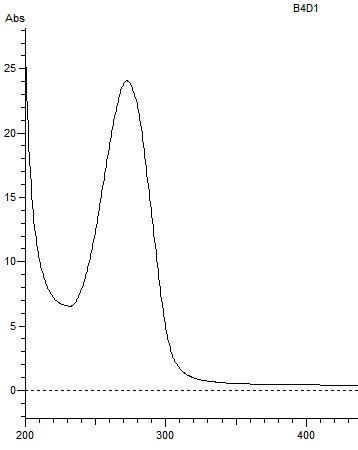


**Figure S40.** UV spectrum of compound **5** in MeOH.


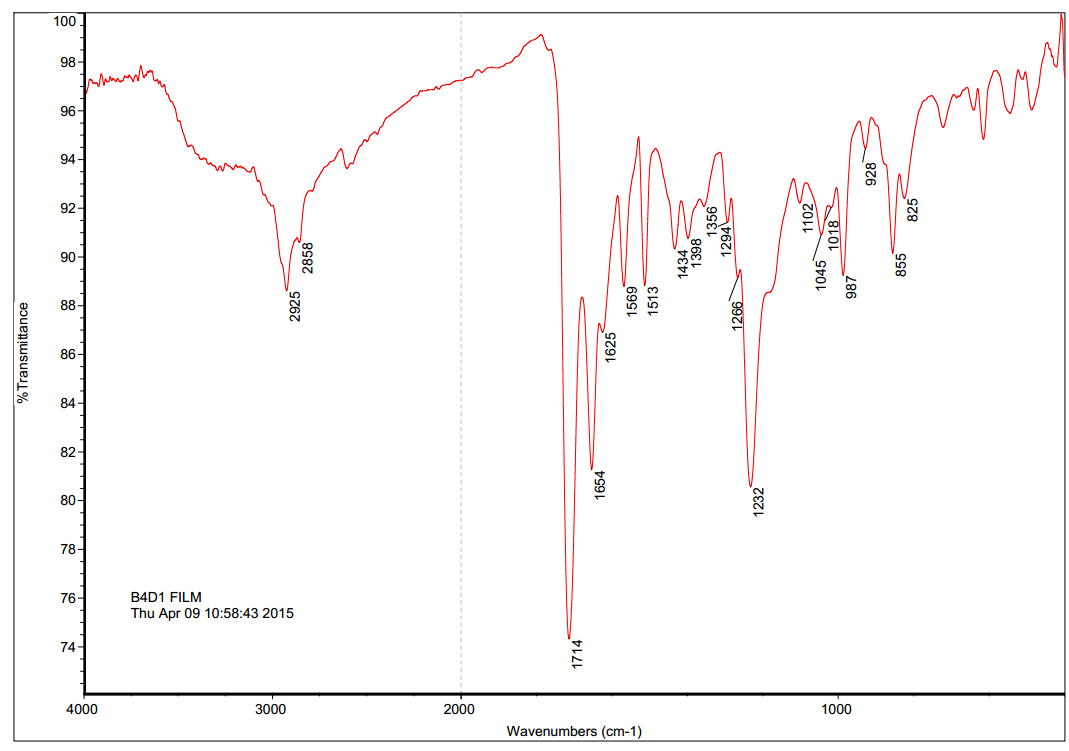


**Figure S41.** IR spectrum of compound **5**.

|  | 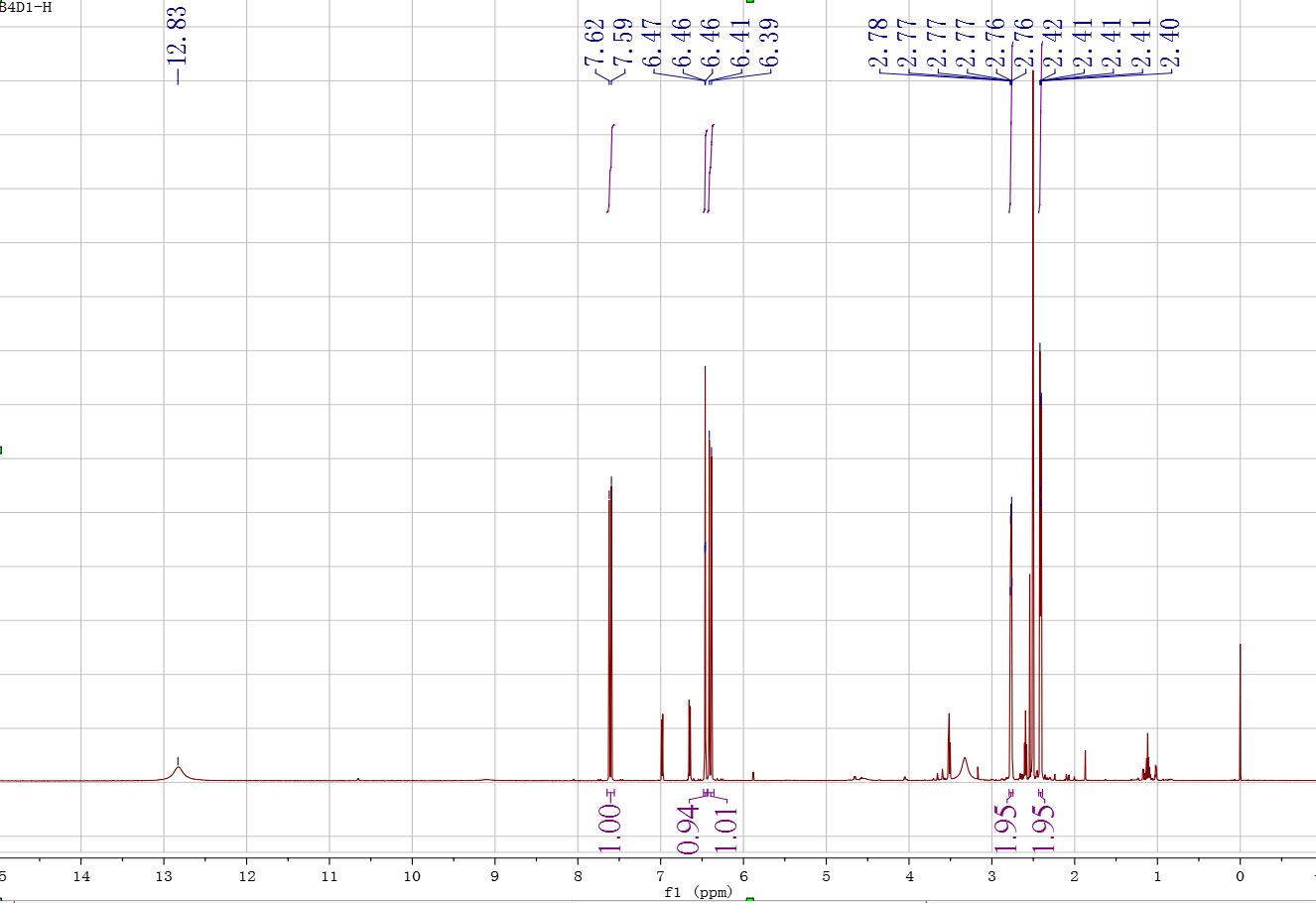 |
| --- | --- |
| (**a**) | (**b**) |

**Figure S42.** (**a**) The structure of compound **5**; (**b**) ^1^H NMR spectrum of compound **5** in DMSO-*d*_6_.


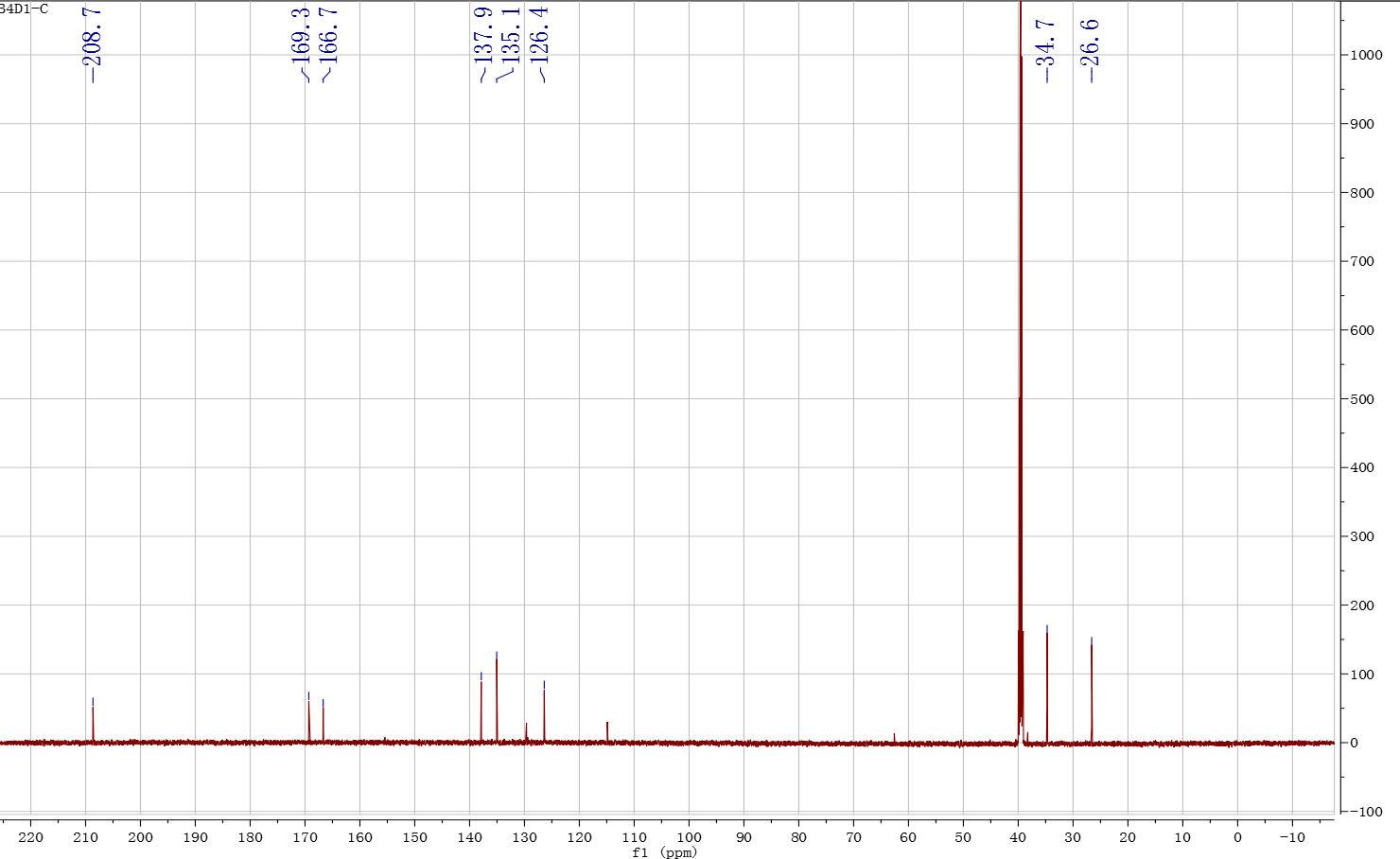


**Figure S43.** ^13^C NMR spectrum of compound **5** in DMSO-*d*_6_.


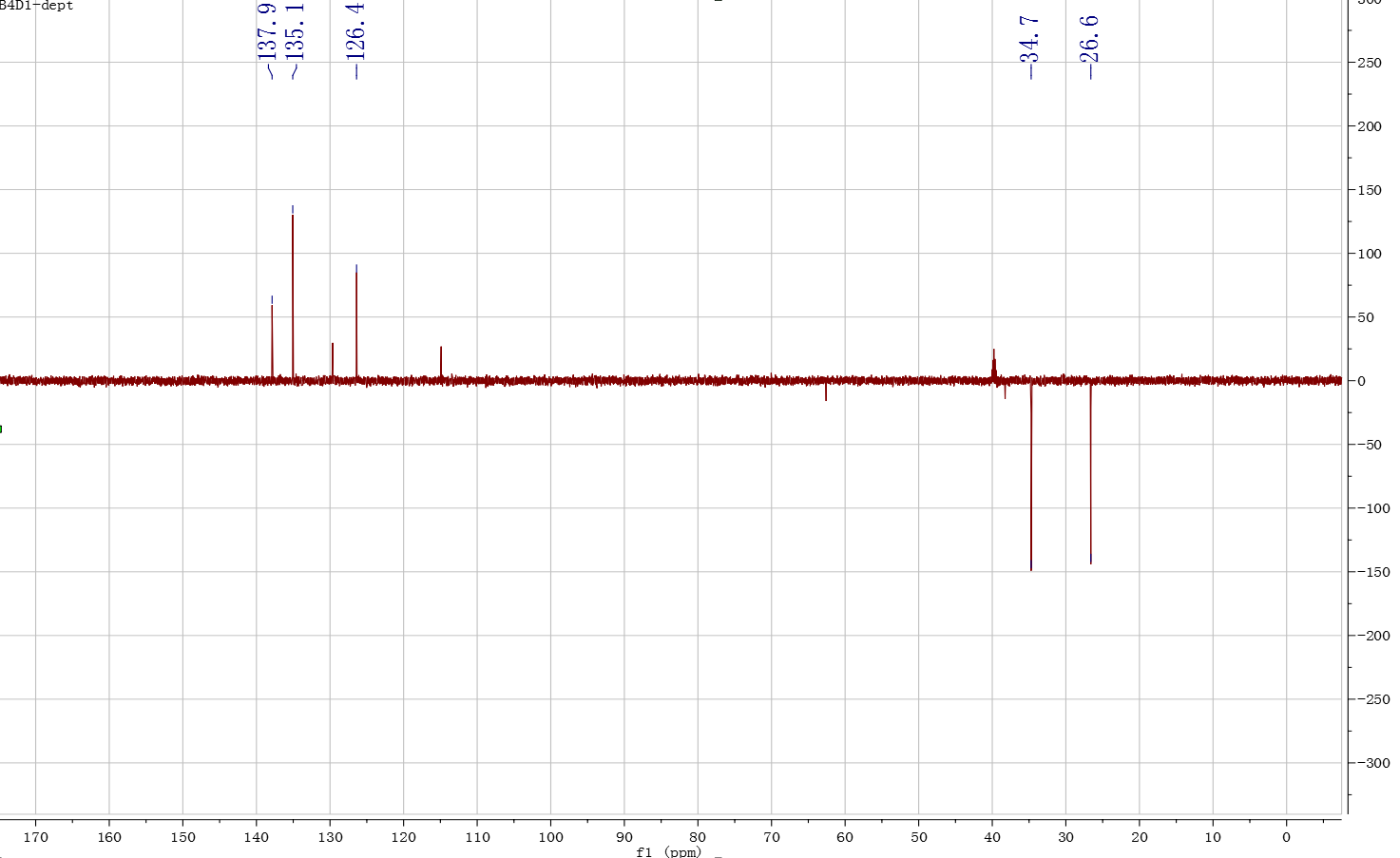


**Figure S44.** DEPT spectrum of compound **5** in DMSO-*d*_6_.


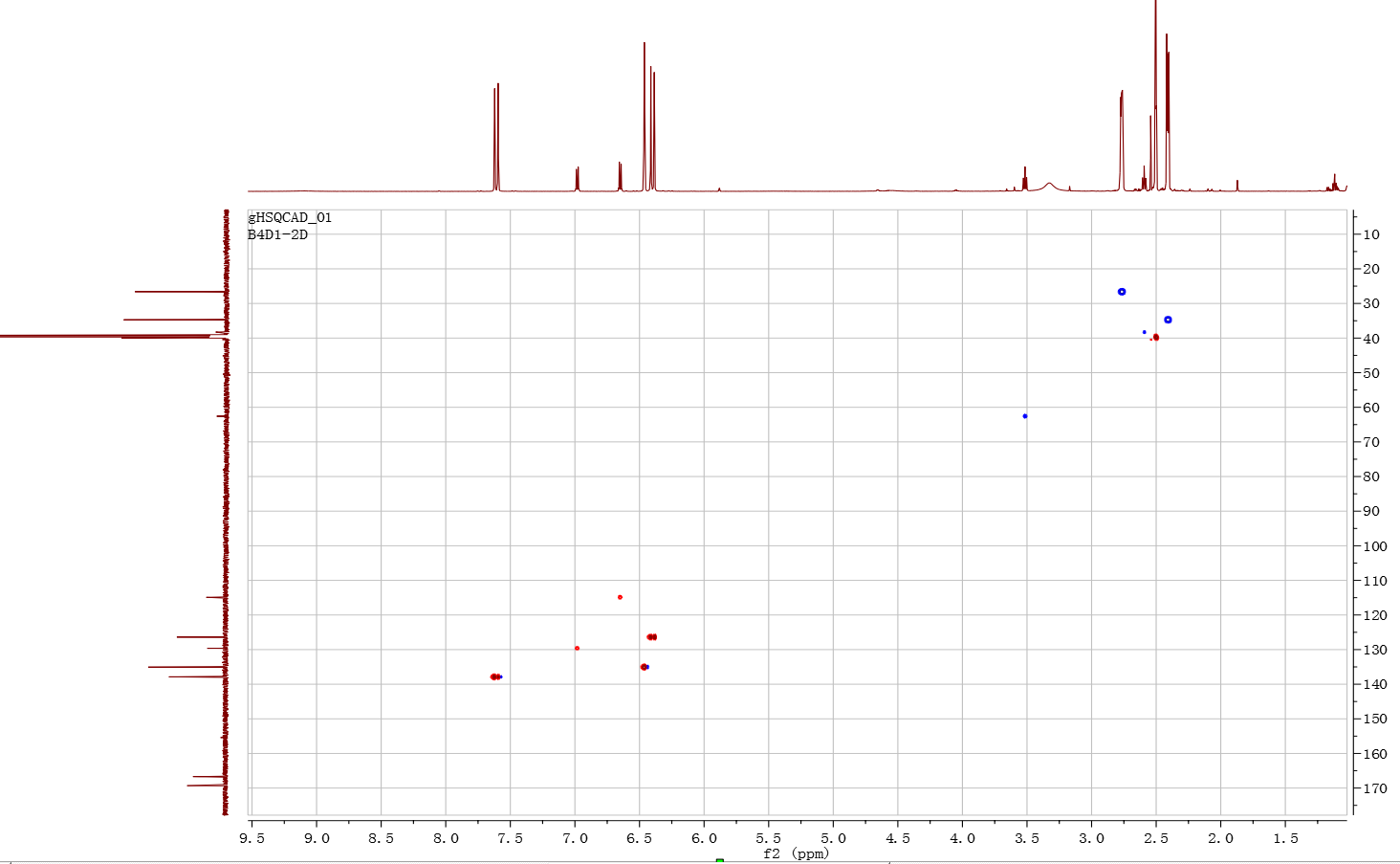


**Figure S45.** HSQC spectrum of compound **5** in DMSO-*d*_6_.


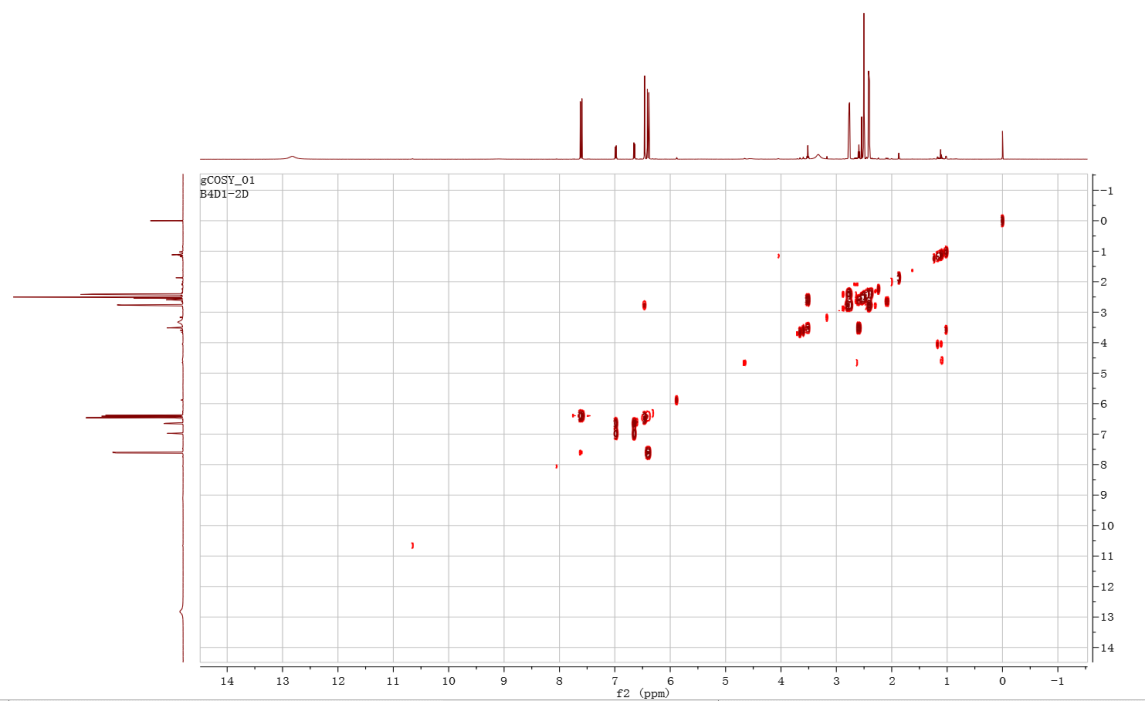


**Figure S46.** ^1^H-^1^H COSY spectrum of compound **5** in DMSO-*d*_6_.


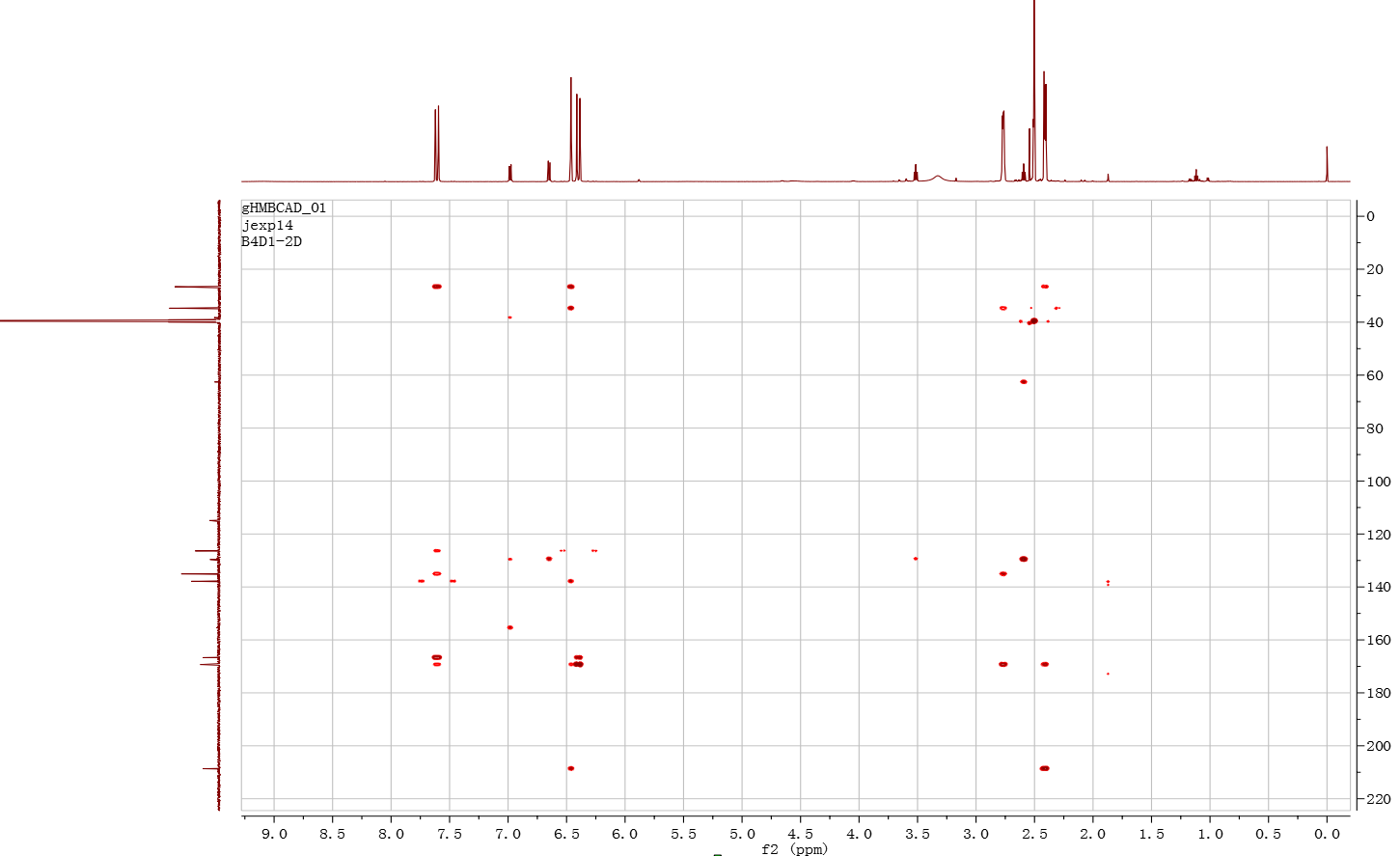


**Figure S47.** HMBC spectrum of compound **5** in DMSO-*d*_6_.

References

1. MacroModel, Schrödinger LLC, 2012. Available online: http://www.schrodinger.com/
   productpage/14/11/ (accessed on 25 November 2014).
2. Frisch, M.J.; Trucks, G.W.; Schlegel, H.B.; Scuseria, G.E.; Robb, M.A.; Cheeseman, J.R.; Scalmani, G.; Barone, V.; Mennucci, B.; Petersson, G.A.; *et al*. *Gaussian 09*; Revision B.01; Gaussian, Inc.: Wallingford, CT, USA, 2010.
3. Bruhn, T.; Schaumlöffel, A.; Hemberger, Y.; Bringmann, G. *SpecDis*; Version 1.50; University of Wuerzburg: Wuerzburg, Germany, 2010.

© 2015 by the authors; licensee MDPI, Basel, Switzerland. This article is an open access article distributed under the terms and conditions of the Creative Commons Attribution license (http://creativecommons.org/licenses/by/4.0/).
